# Supplementary figures and images for: CSQUiD: an index and non-probability framework for constrained skyline query processing over uncertain data
Source: PeerJ Comput Sci. 2024 Sep 16;10:e2225. doi: 10.7717/peerj-cs.2225 (PMC11639172; doi:10.7717/peerj-cs.2225)

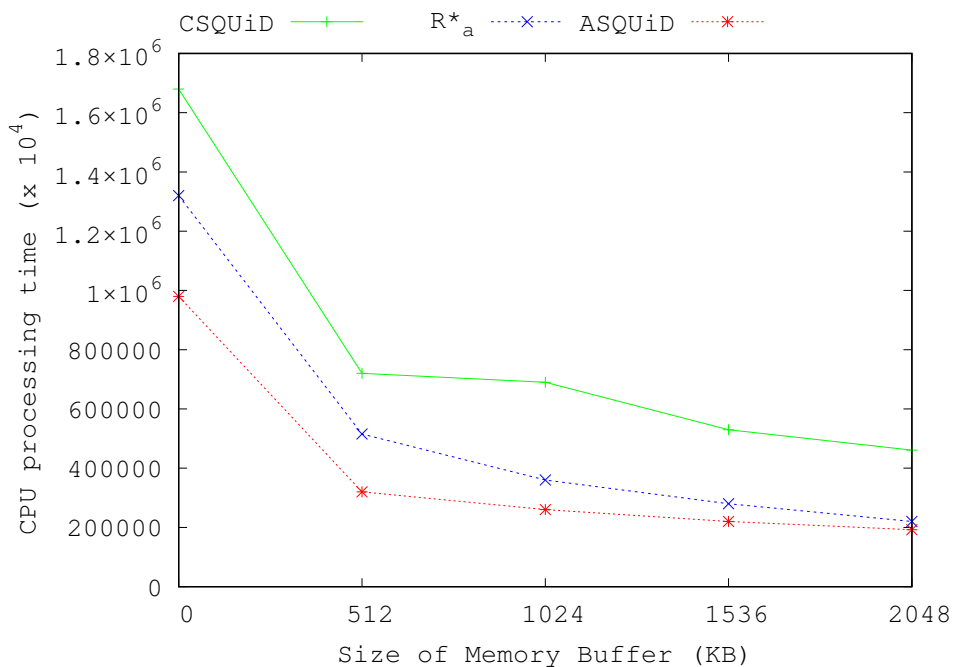

Supplement: Supplemental Information 2 [file peerj-cs-10-2225-s002.zip › Peer-SQUiDExperimentalResults/BS/outputAC-eps-converted-to.pdf]

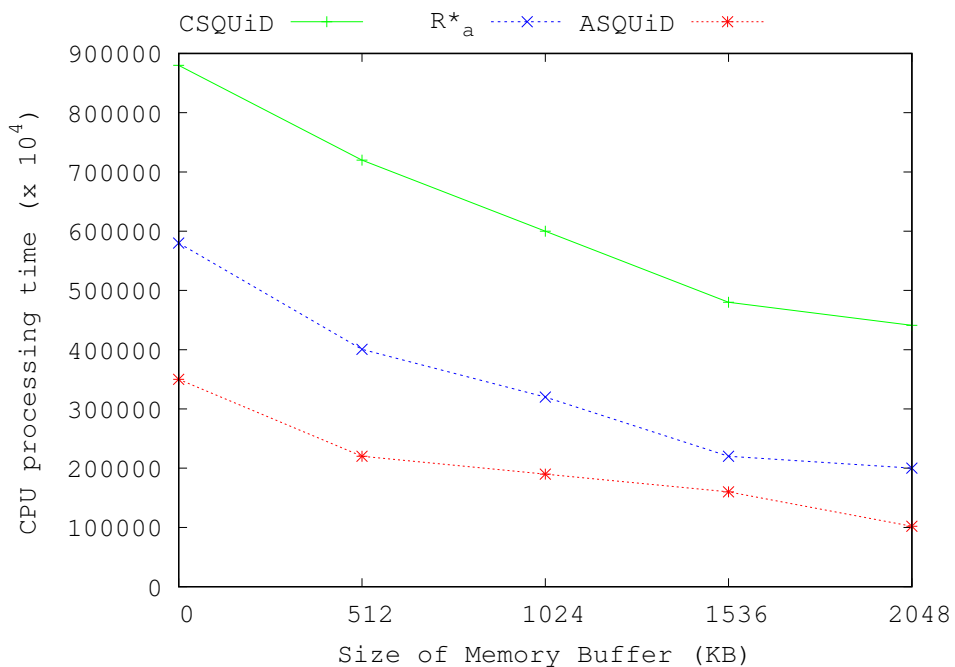

Supplement: Supplemental Information 2 [file peerj-cs-10-2225-s002.zip › Peer-SQUiDExperimentalResults/BS/outputC-eps-converted-to.pdf]

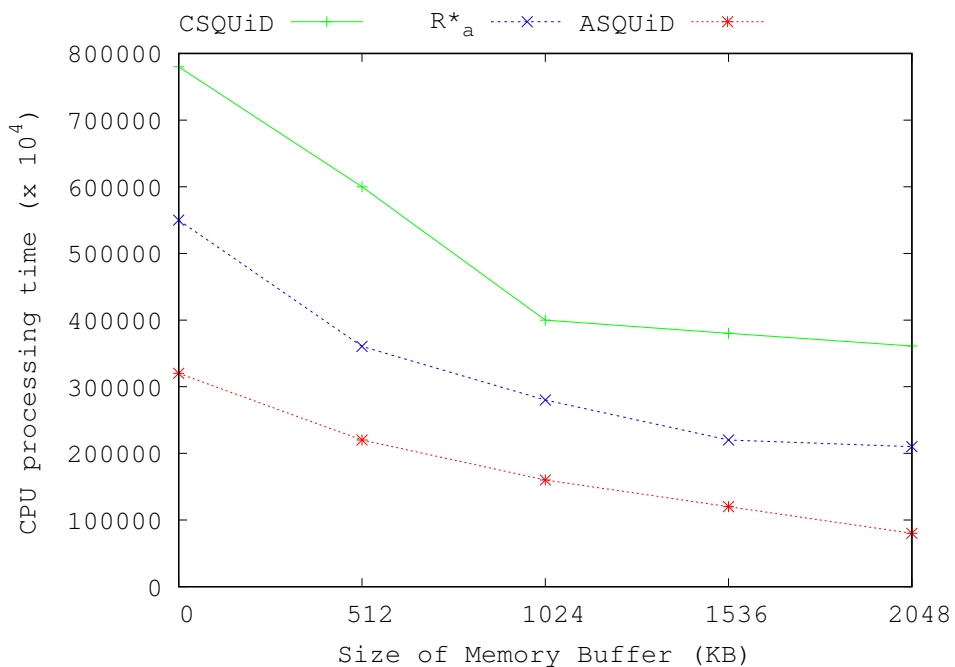

Supplement: Supplemental Information 2 [file peerj-cs-10-2225-s002.zip › Peer-SQUiDExperimentalResults/BS/outputI-eps-converted-to.pdf]

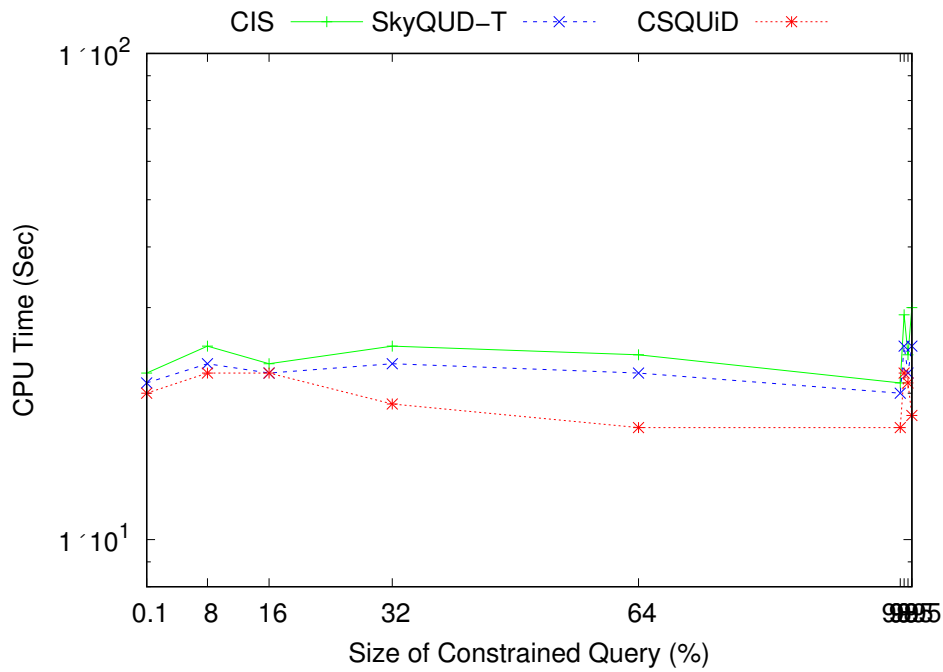

Supplement: Supplemental Information 2 [file peerj-cs-10-2225-s002.zip › Peer-SQUiDExperimentalResults/CQ/CQCPU/outputAC-eps-converted-to.pdf]

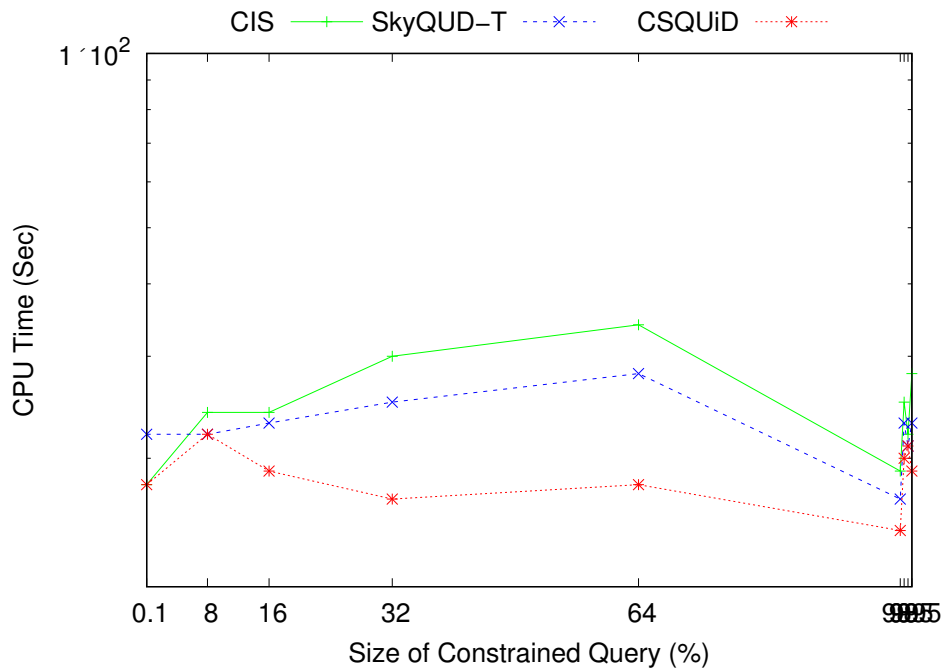

Supplement: Supplemental Information 2 [file peerj-cs-10-2225-s002.zip › Peer-SQUiDExperimentalResults/CQ/CQCPU/outputC-eps-converted-to.pdf]

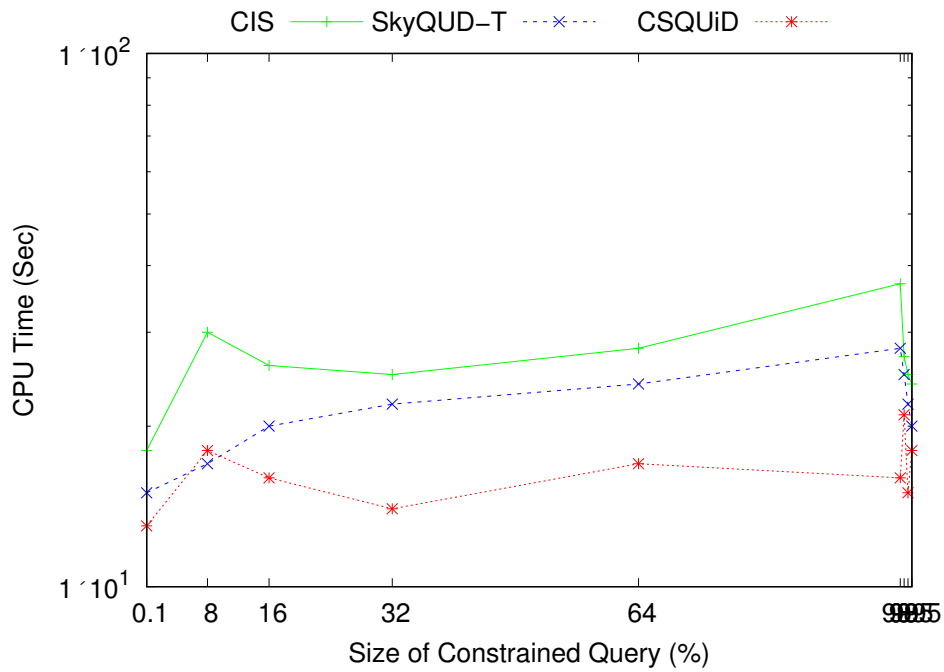

Supplement: Supplemental Information 2 [file peerj-cs-10-2225-s002.zip › Peer-SQUiDExperimentalResults/CQ/CQCPU/outputI-eps-converted-to.pdf]

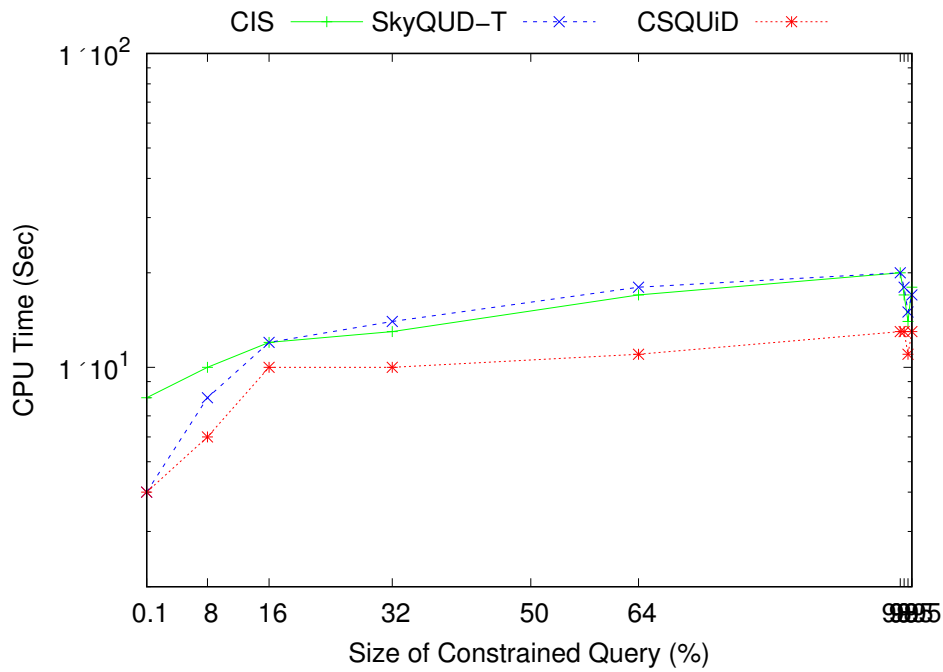

Supplement: Supplemental Information 2 [file peerj-cs-10-2225-s002.zip › Peer-SQUiDExperimentalResults/CQ/CQCPU/outputN-eps-converted-to.pdf]

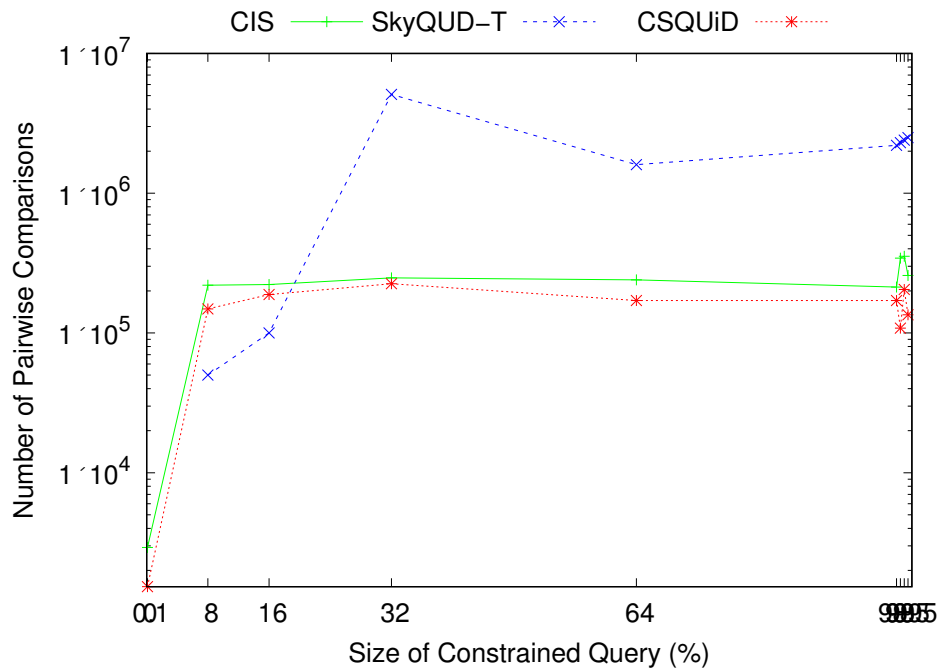

Supplement: Supplemental Information 2 [file peerj-cs-10-2225-s002.zip › Peer-SQUiDExperimentalResults/CQ/CQNNV/outputAC-eps-converted-to.pdf]

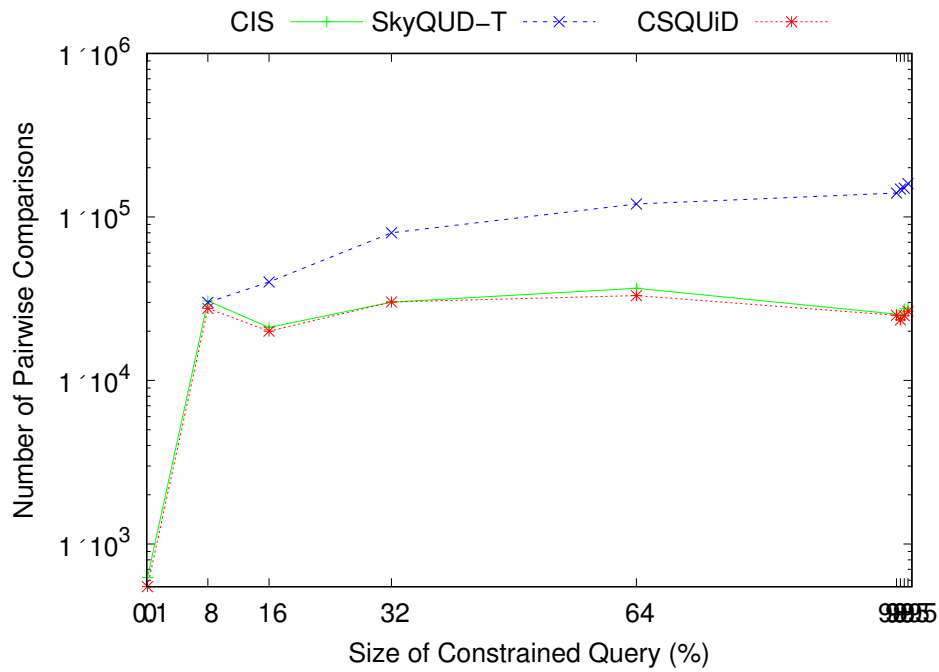

Supplement: Supplemental Information 2 [file peerj-cs-10-2225-s002.zip › Peer-SQUiDExperimentalResults/CQ/CQNNV/outputC-eps-converted-to.pdf]

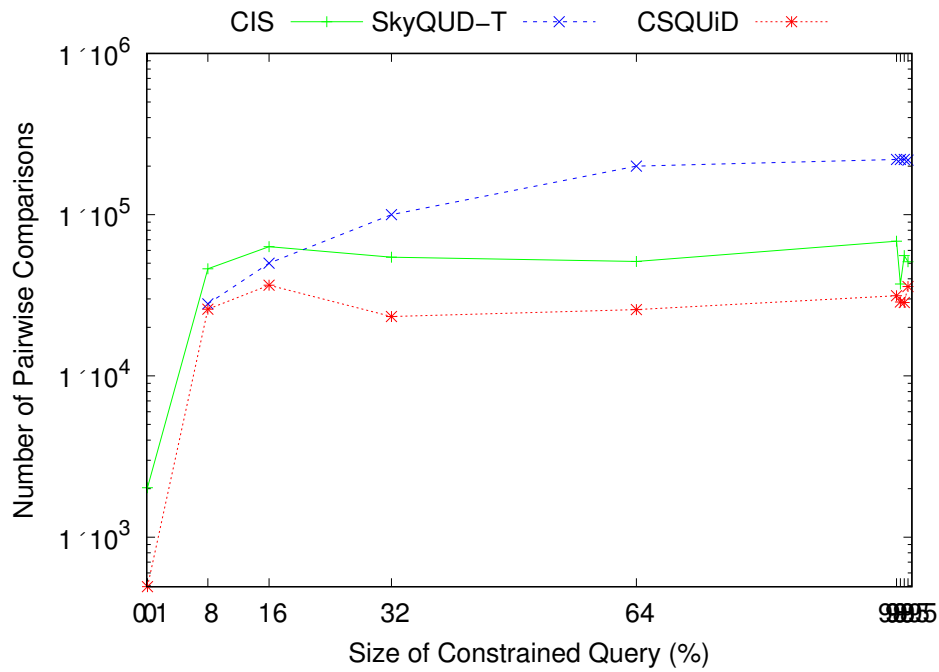

Supplement: Supplemental Information 2 [file peerj-cs-10-2225-s002.zip › Peer-SQUiDExperimentalResults/CQ/CQNNV/outputI-eps-converted-to.pdf]

Number of Pairwise Comparisons

CIS —+— SkyQUD-T - - × - - CSQUD - - \* - -

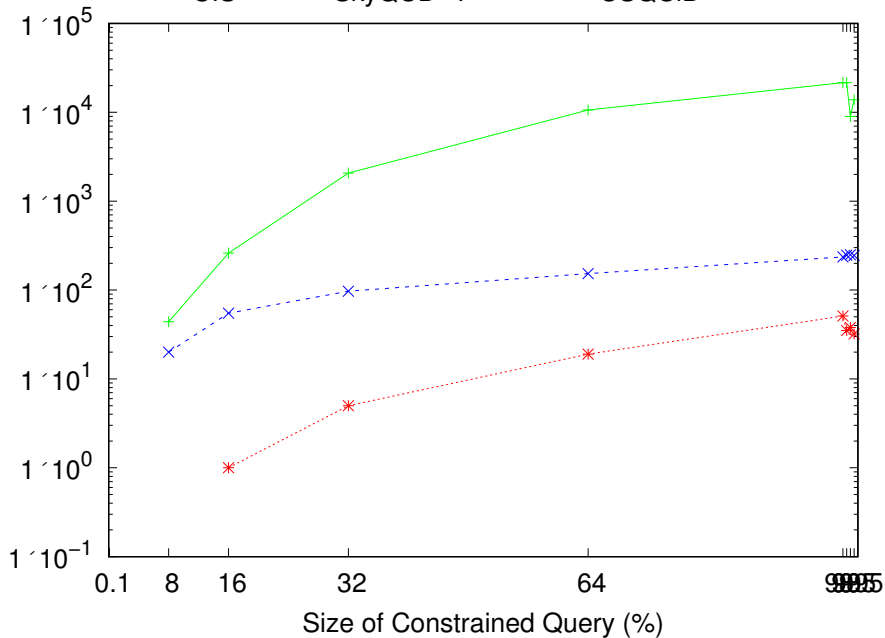

Supplement: Supplemental Information 2 [file peerj-cs-10-2225-s002.zip › Peer-SQUiDExperimentalResults/CQ/CQNNV/outputN-eps-converted-to.pdf]

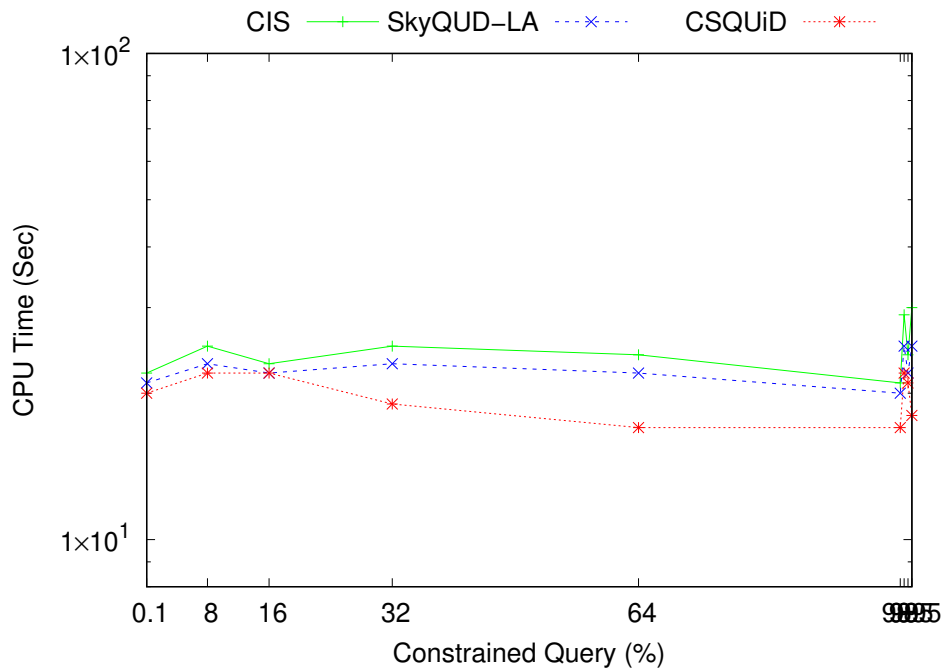

Supplement: Supplemental Information 2 [file peerj-cs-10-2225-s002.zip › Peer-SQUiDExperimentalResults/CQ1/CQCPU/outputAC-eps-converted-to.pdf]

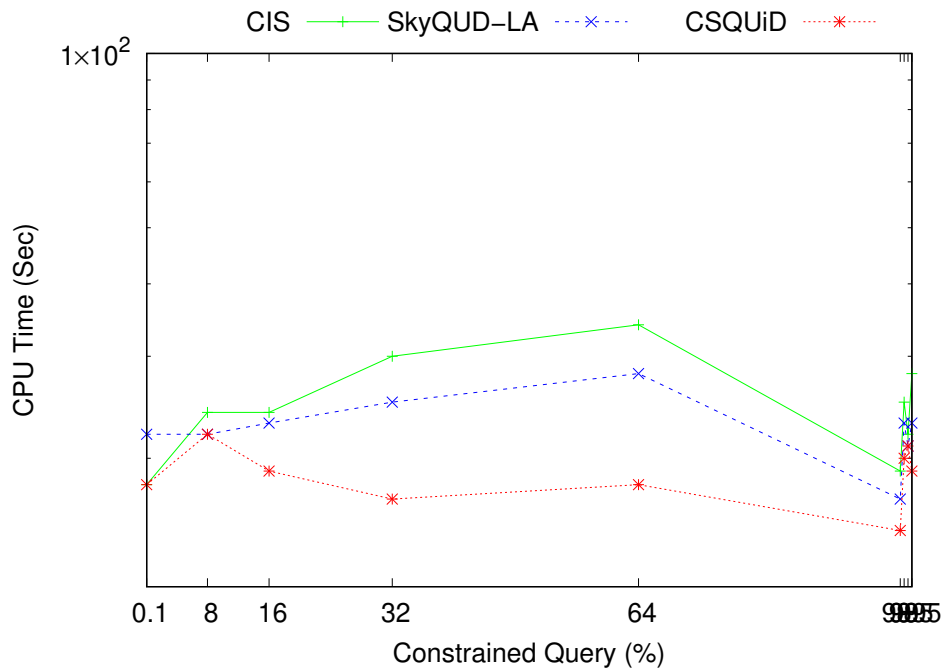

Supplement: Supplemental Information 2 [file peerj-cs-10-2225-s002.zip › Peer-SQUiDExperimentalResults/CQ1/CQCPU/outputC-eps-converted-to.pdf]

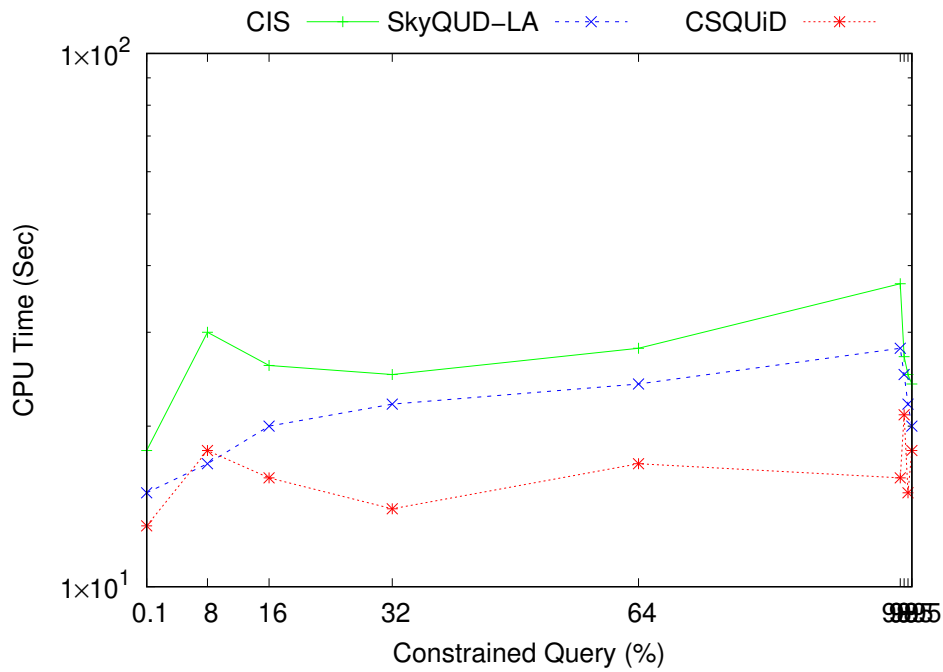

Supplement: Supplemental Information 2 [file peerj-cs-10-2225-s002.zip › Peer-SQUiDExperimentalResults/CQ1/CQCPU/outputI-eps-converted-to.pdf]

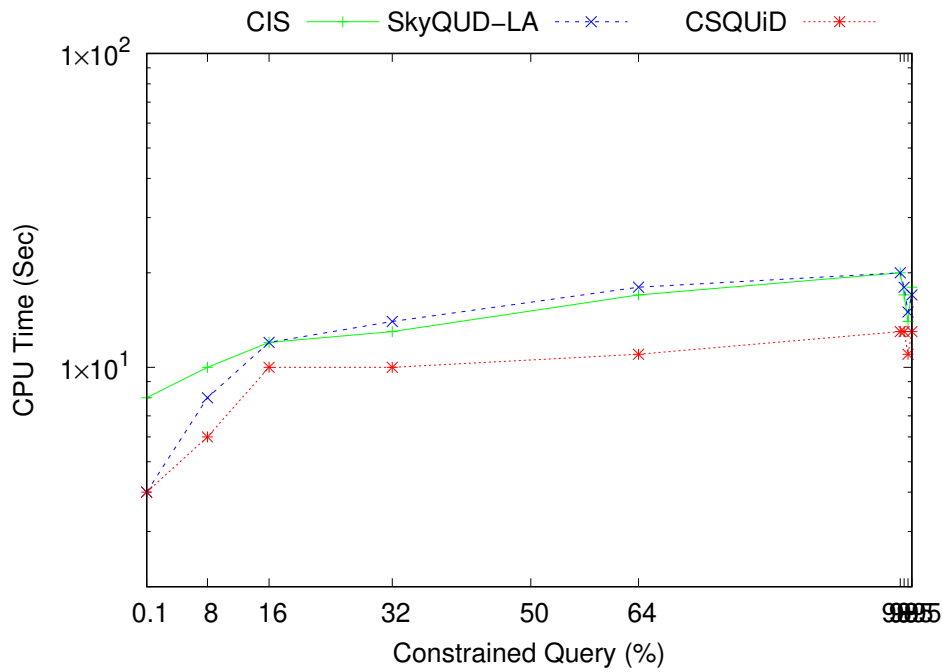

Supplement: Supplemental Information 2 [file peerj-cs-10-2225-s002.zip › Peer-SQUiDExperimentalResults/CQ1/CQCPU/outputN-eps-converted-to.pdf]

Number of Pairwise Comparisons

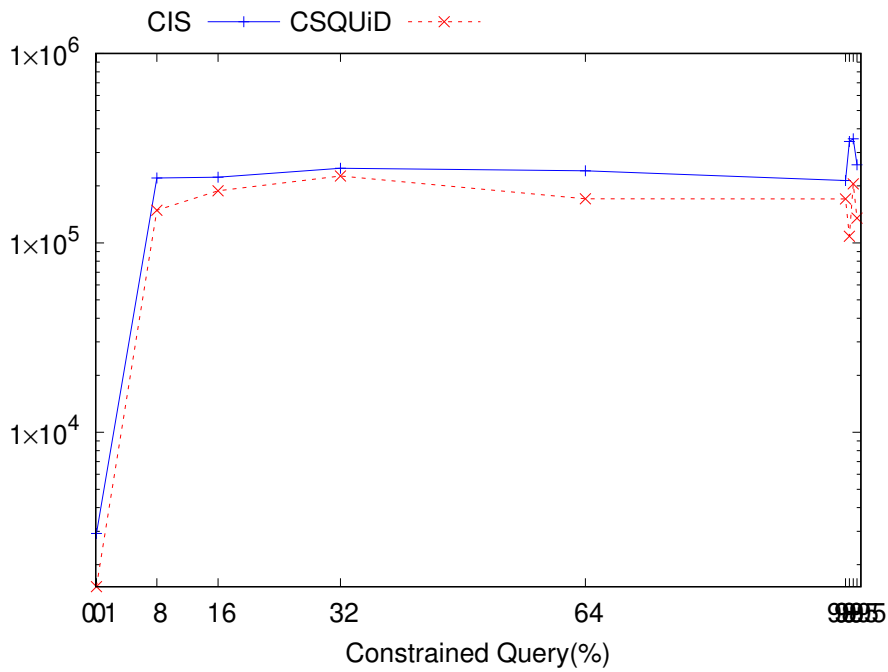

Supplement: Supplemental Information 2 [file peerj-cs-10-2225-s002.zip › Peer-SQUiDExperimentalResults/CQ1/CQNNV/outputAC-eps-converted-to.pdf]

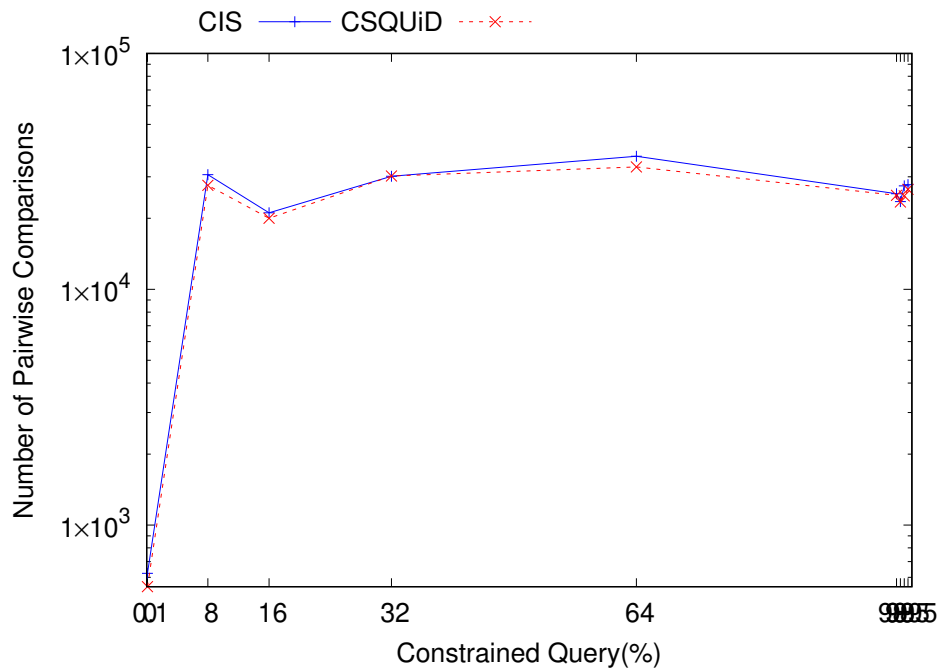

Supplement: Supplemental Information 2 [file peerj-cs-10-2225-s002.zip › Peer-SQUiDExperimentalResults/CQ1/CQNNV/outputC-eps-converted-to.pdf]

Number of Pairwise Comparisons

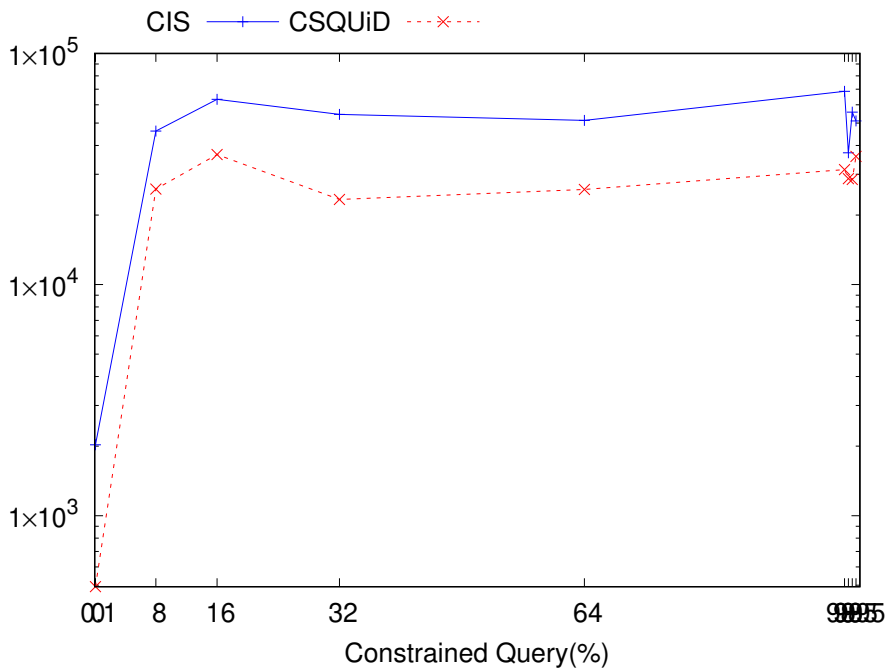

Supplement: Supplemental Information 2 [file peerj-cs-10-2225-s002.zip › Peer-SQUiDExperimentalResults/CQ1/CQNNV/outputI-eps-converted-to.pdf]

Number of Pairwise Comparisons

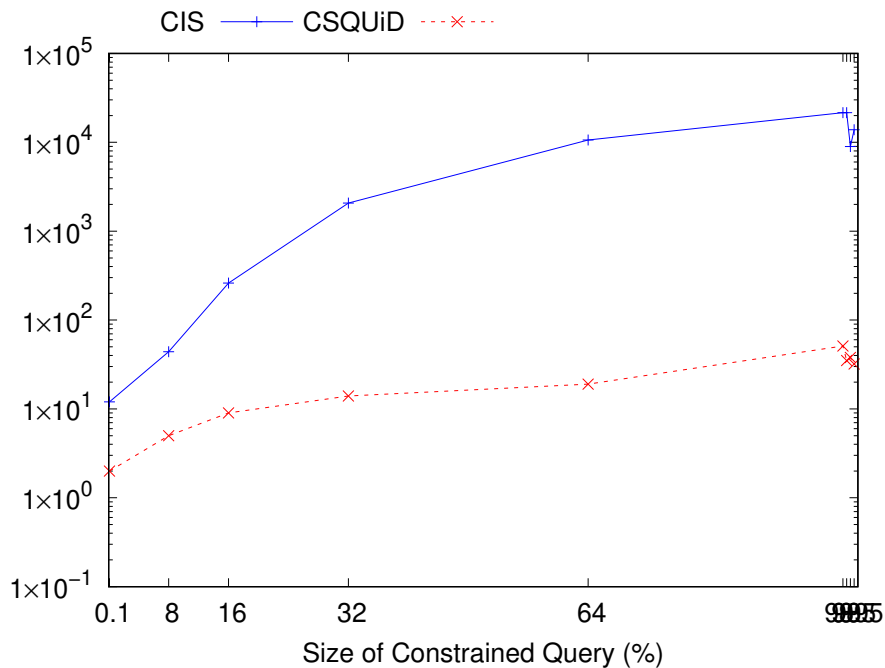

Supplement: Supplemental Information 2 [file peerj-cs-10-2225-s002.zip › Peer-SQUiDExperimentalResults/CQ1/CQNNV/outputN-eps-converted-to.pdf]

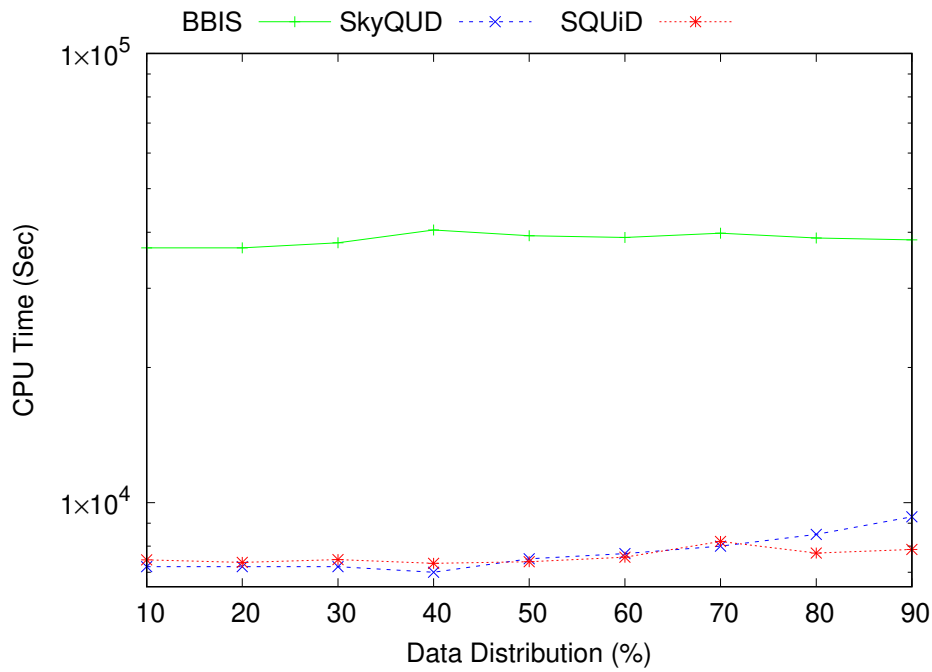

Supplement: Supplemental Information 2 [file peerj-cs-10-2225-s002.zip › Peer-SQUiDExperimentalResults/DD/DDCPU/outputAC-eps-converted-to.pdf]

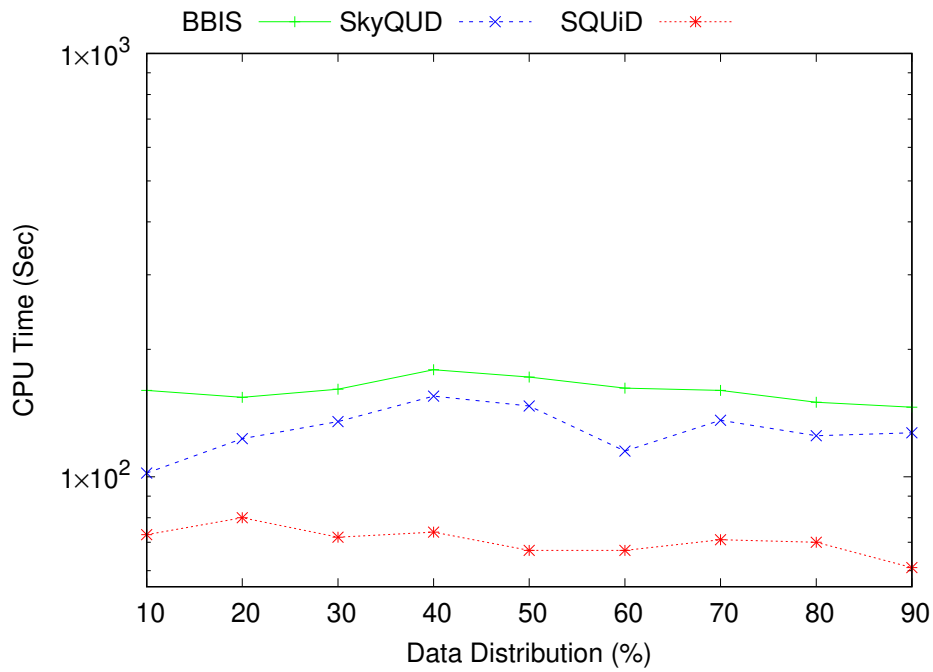

Supplement: Supplemental Information 2 [file peerj-cs-10-2225-s002.zip › Peer-SQUiDExperimentalResults/DD/DDCPU/outputC-eps-converted-to.pdf]

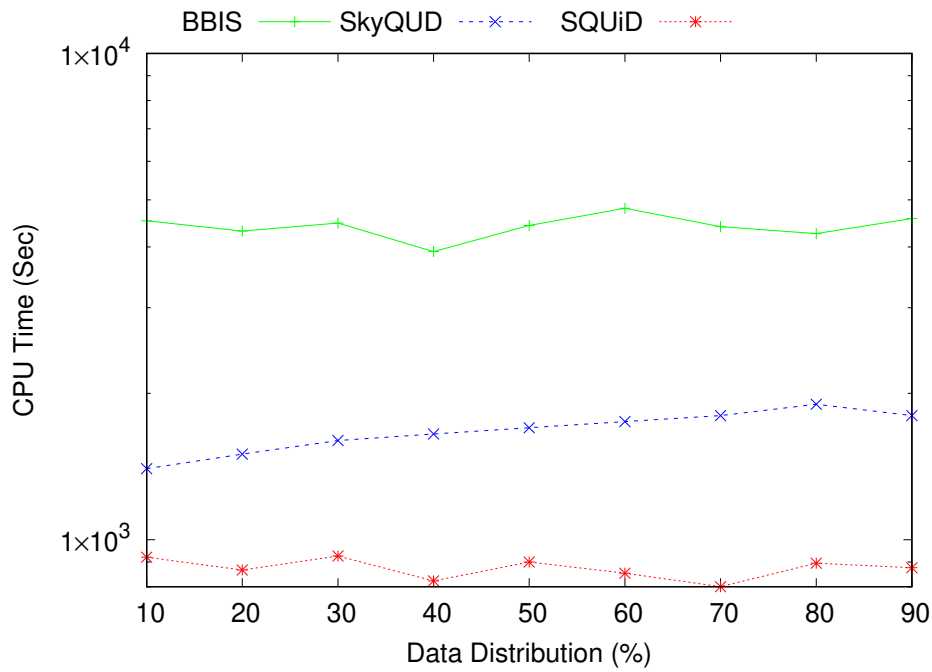

Supplement: Supplemental Information 2 [file peerj-cs-10-2225-s002.zip › Peer-SQUiDExperimentalResults/DD/DDCPU/outputI-eps-converted-to.pdf]

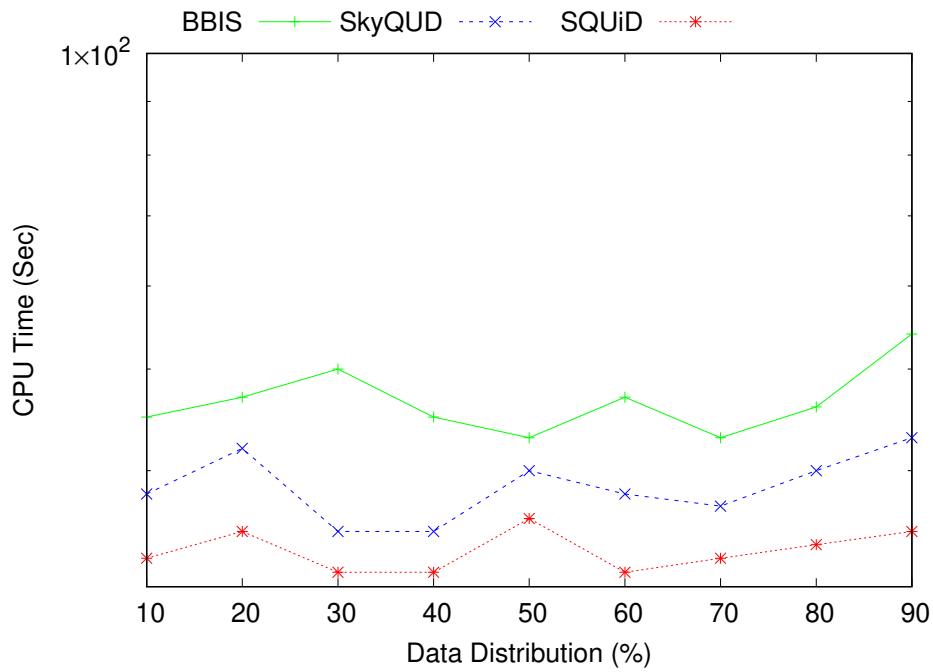

Supplement: Supplemental Information 2 [file peerj-cs-10-2225-s002.zip › Peer-SQUiDExperimentalResults/DD/DDCPU/outputN-eps-converted-to.pdf]

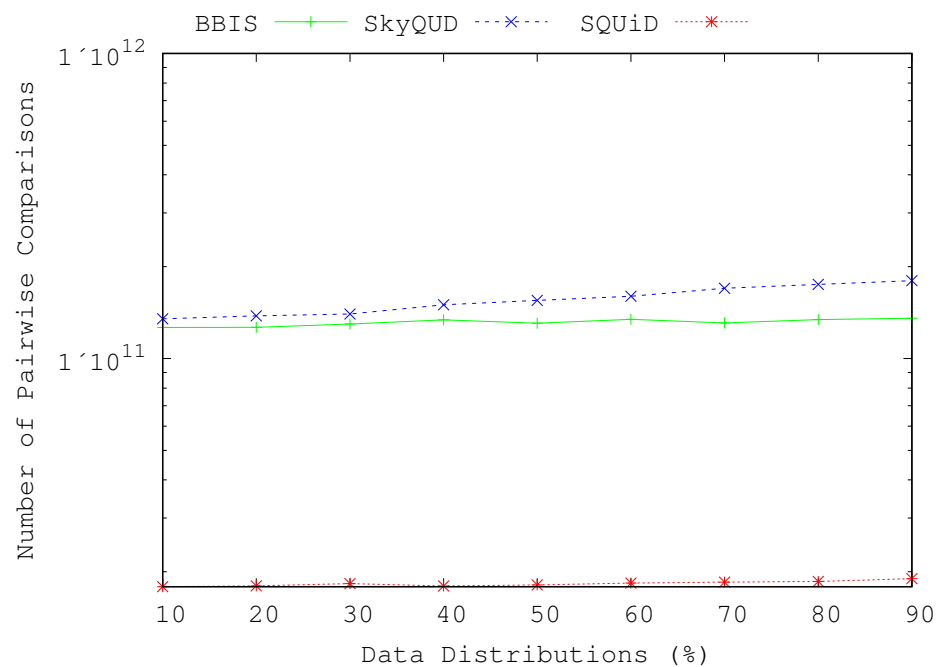

Supplement: Supplemental Information 2 [file peerj-cs-10-2225-s002.zip › Peer-SQUiDExperimentalResults/DD/DDNNV/outputAC-eps-converted-to.pdf]

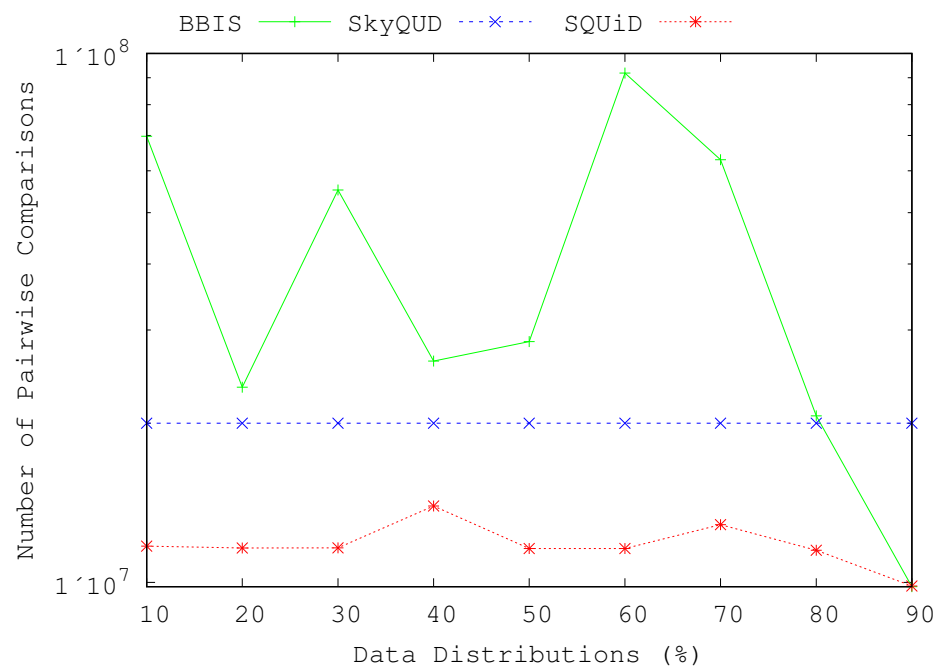

Supplement: Supplemental Information 2 [file peerj-cs-10-2225-s002.zip › Peer-SQUiDExperimentalResults/DD/DDNNV/outputC-eps-converted-to.pdf]

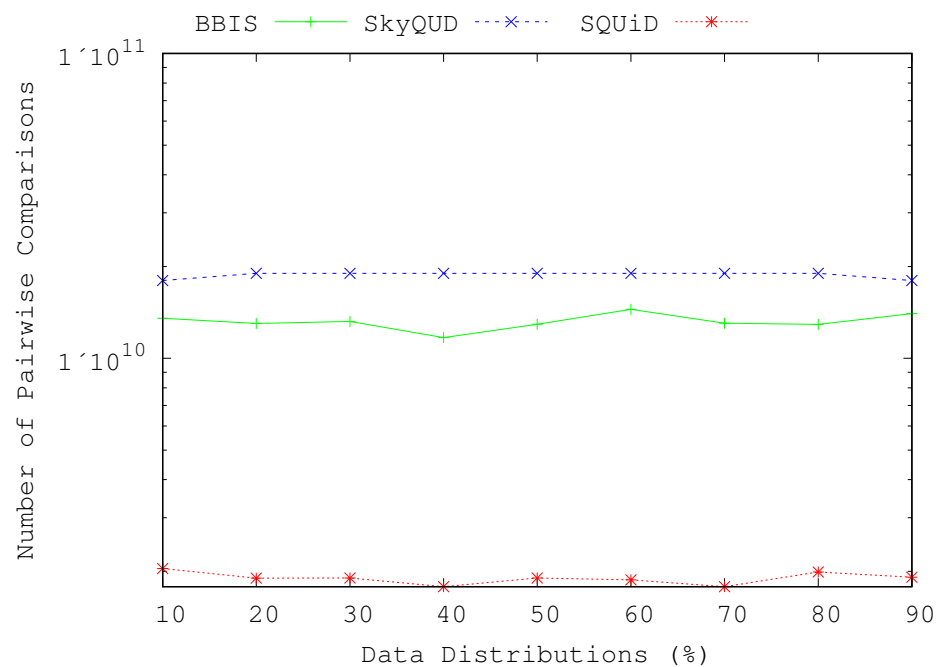

Supplement: Supplemental Information 2 [file peerj-cs-10-2225-s002.zip › Peer-SQUiDExperimentalResults/DD/DDNNV/outputI-eps-converted-to.pdf]

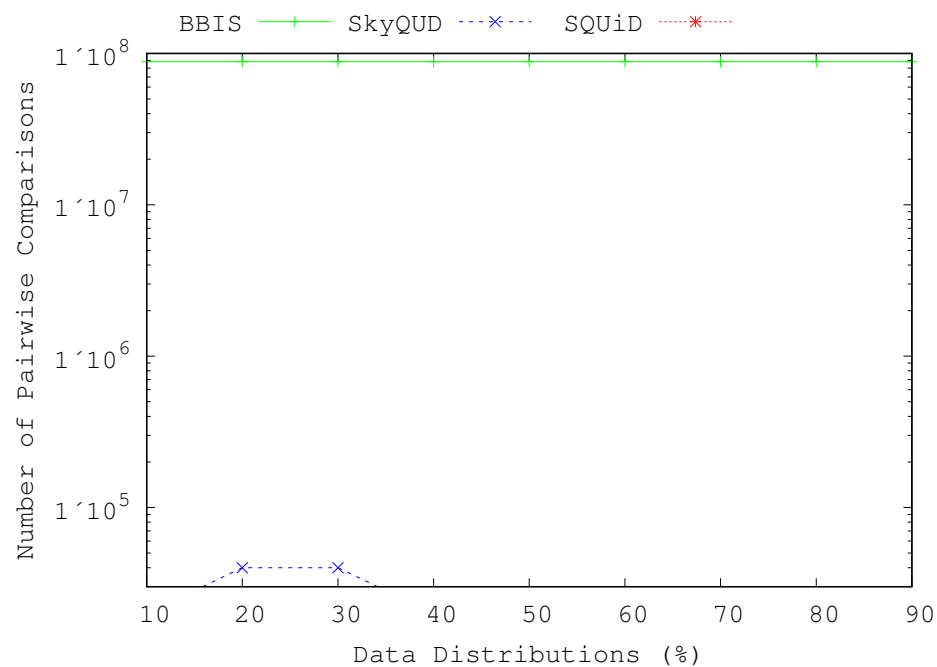

Supplement: Supplemental Information 2 [file peerj-cs-10-2225-s002.zip › Peer-SQUiDExperimentalResults/DD/DDNNV/outputN-eps-converted-to.pdf]

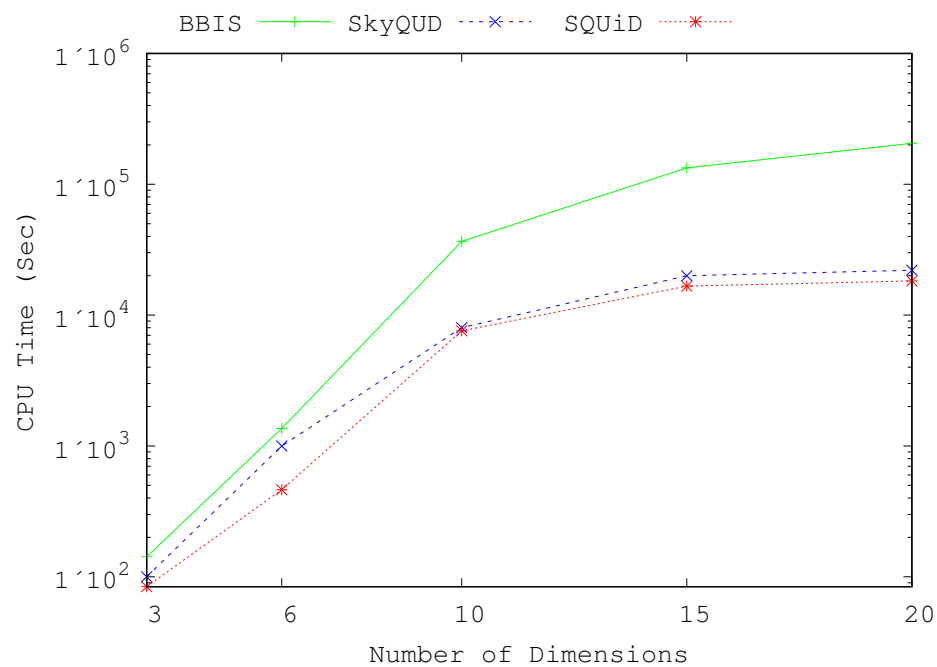

Supplement: Supplemental Information 2 [file peerj-cs-10-2225-s002.zip › Peer-SQUiDExperimentalResults/DM/DMCPU/outputAC-eps-converted-to.pdf]

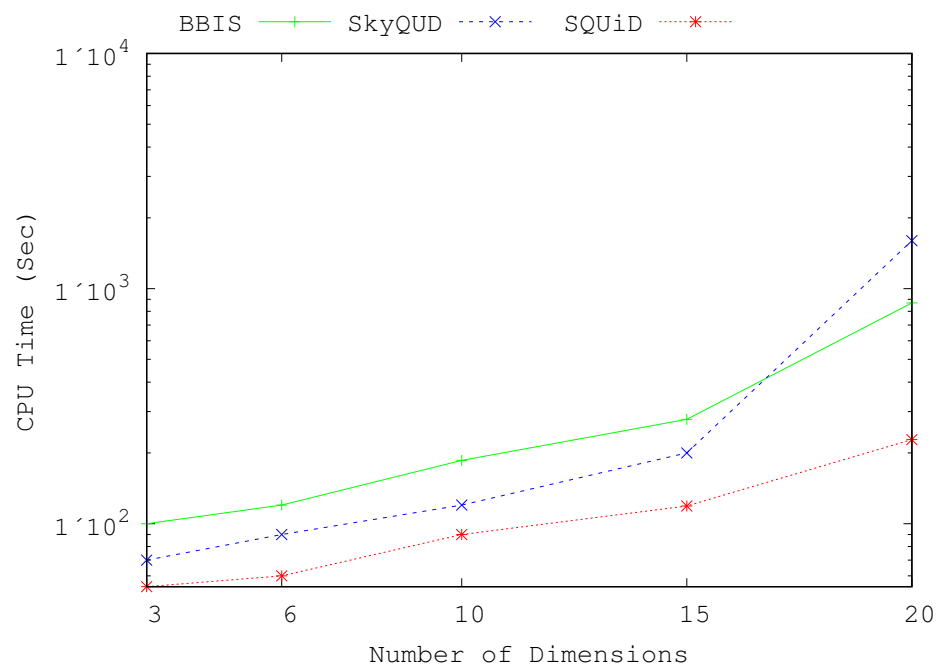

Supplement: Supplemental Information 2 [file peerj-cs-10-2225-s002.zip › Peer-SQUiDExperimentalResults/DM/DMCPU/outputC-eps-converted-to.pdf]

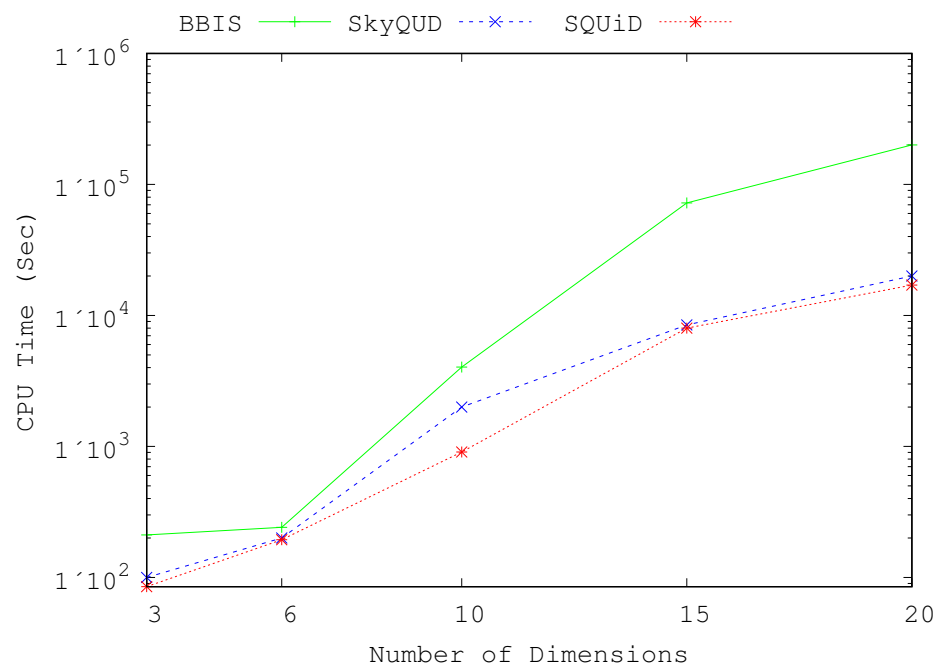

Supplement: Supplemental Information 2 [file peerj-cs-10-2225-s002.zip › Peer-SQUiDExperimentalResults/DM/DMCPU/outputI-eps-converted-to.pdf]

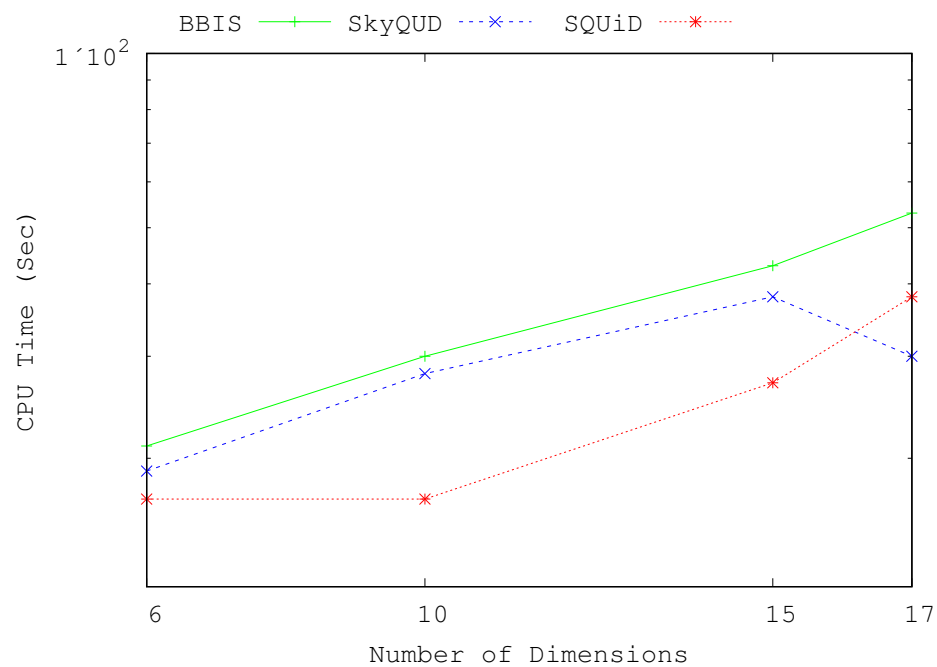

Supplement: Supplemental Information 2 [file peerj-cs-10-2225-s002.zip › Peer-SQUiDExperimentalResults/DM/DMCPU/outputN-eps-converted-to.pdf]

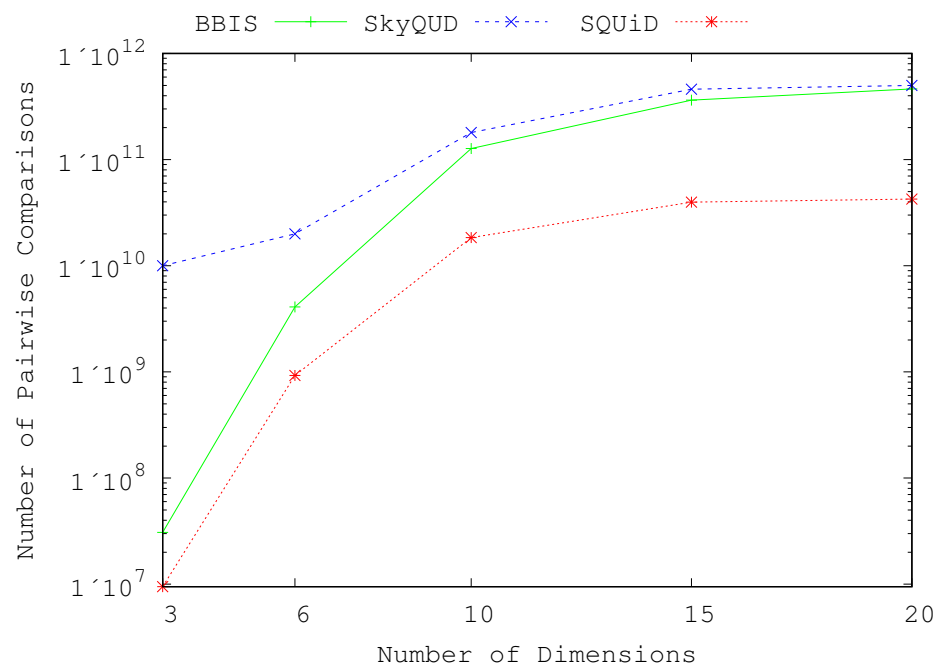

Supplement: Supplemental Information 2 [file peerj-cs-10-2225-s002.zip › Peer-SQUiDExperimentalResults/DM/DMNNV/outputAC-eps-converted-to.pdf]

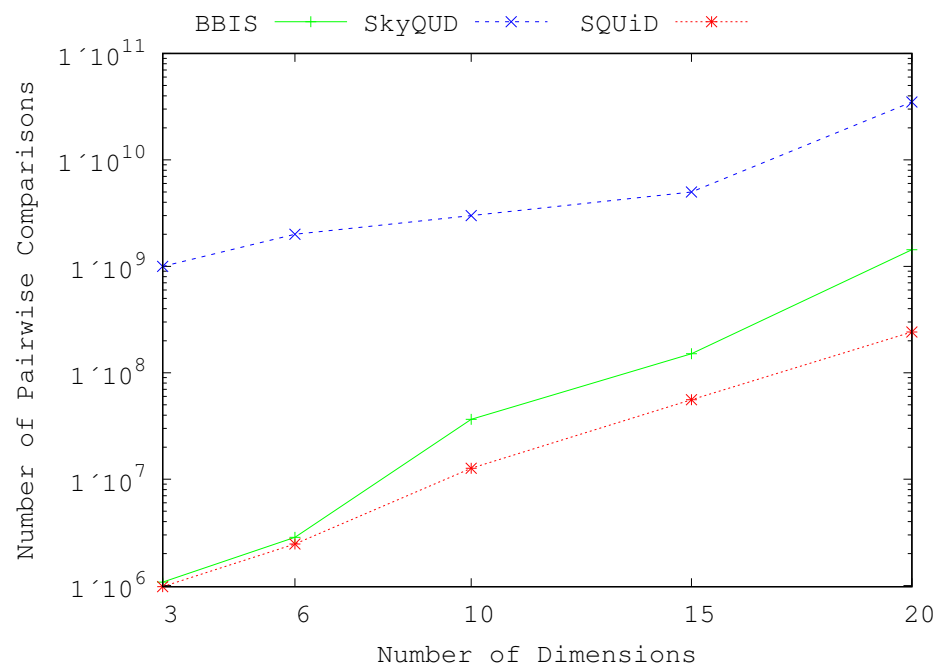

Supplement: Supplemental Information 2 [file peerj-cs-10-2225-s002.zip › Peer-SQUiDExperimentalResults/DM/DMNNV/outputC-eps-converted-to.pdf]

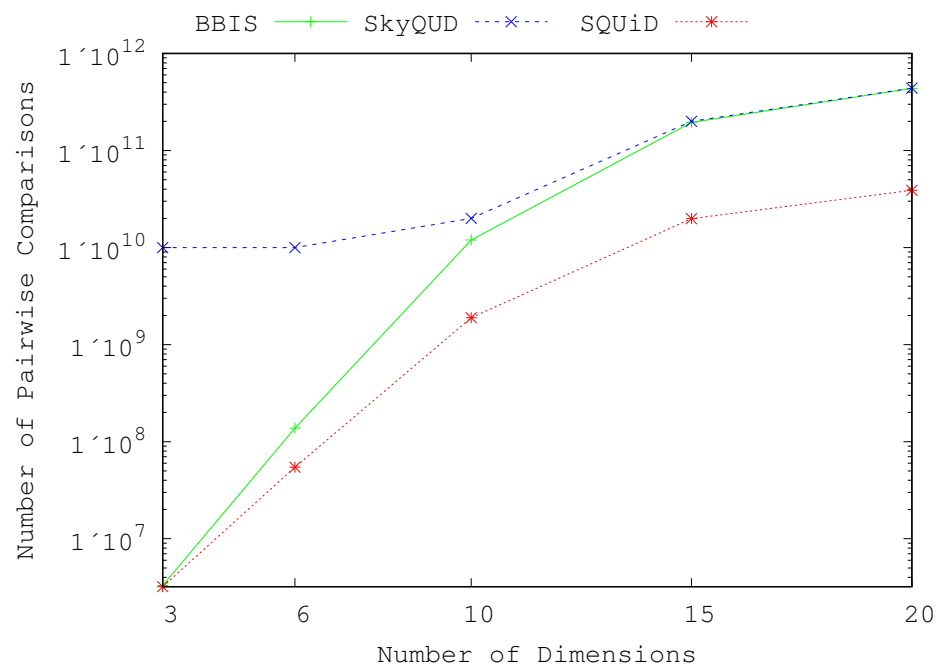

Supplement: Supplemental Information 2 [file peerj-cs-10-2225-s002.zip › Peer-SQUiDExperimentalResults/DM/DMNNV/outputI-eps-converted-to.pdf]

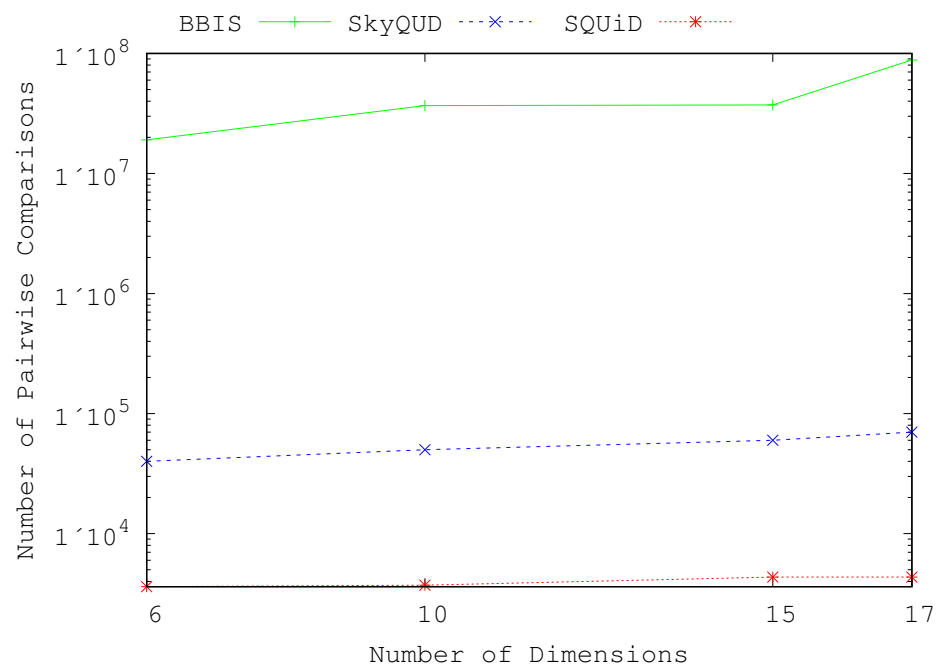

Supplement: Supplemental Information 2 [file peerj-cs-10-2225-s002.zip › Peer-SQUiDExperimentalResults/DM/DMNNV/outputN-eps-converted-to.pdf]

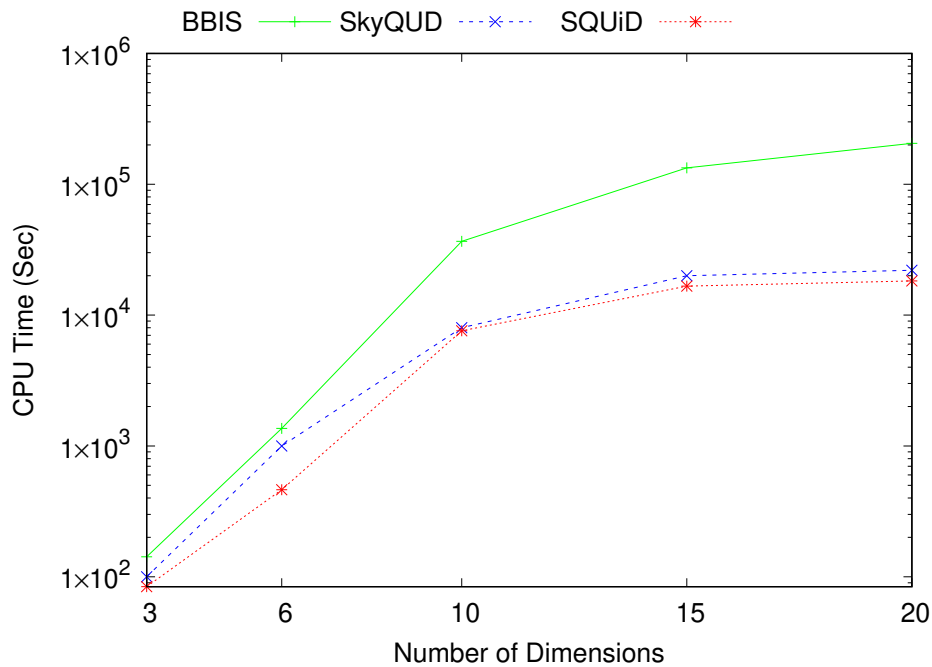

Supplement: Supplemental Information 2 [file peerj-cs-10-2225-s002.zip › Peer-SQUiDExperimentalResults/DM1/DMCPU/outputAC-eps-converted-to.pdf]

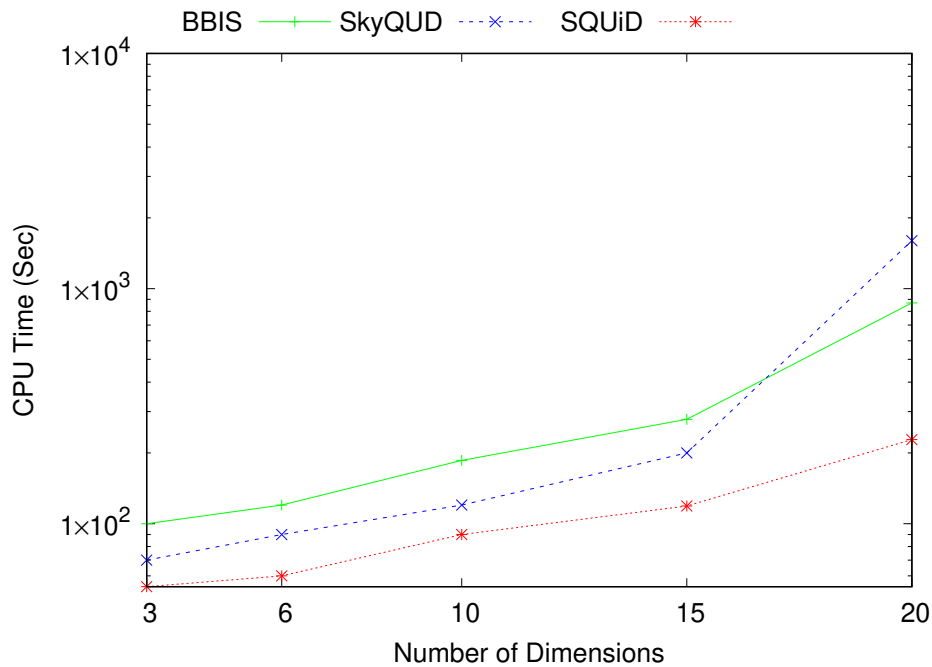

Supplement: Supplemental Information 2 [file peerj-cs-10-2225-s002.zip › Peer-SQUiDExperimentalResults/DM1/DMCPU/outputC-eps-converted-to.pdf]

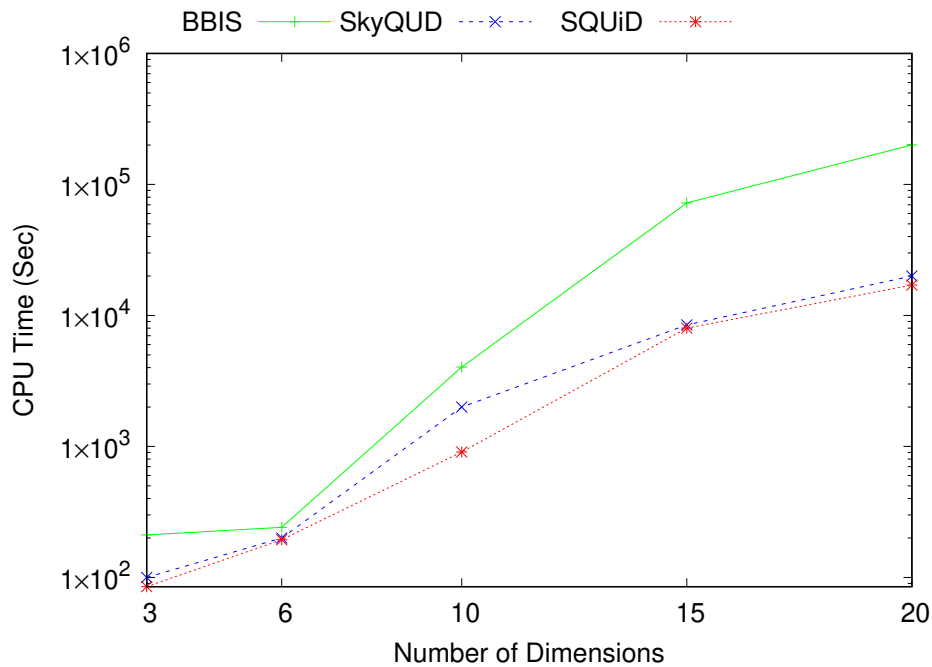

Supplement: Supplemental Information 2 [file peerj-cs-10-2225-s002.zip › Peer-SQUiDExperimentalResults/DM1/DMCPU/outputI-eps-converted-to.pdf]

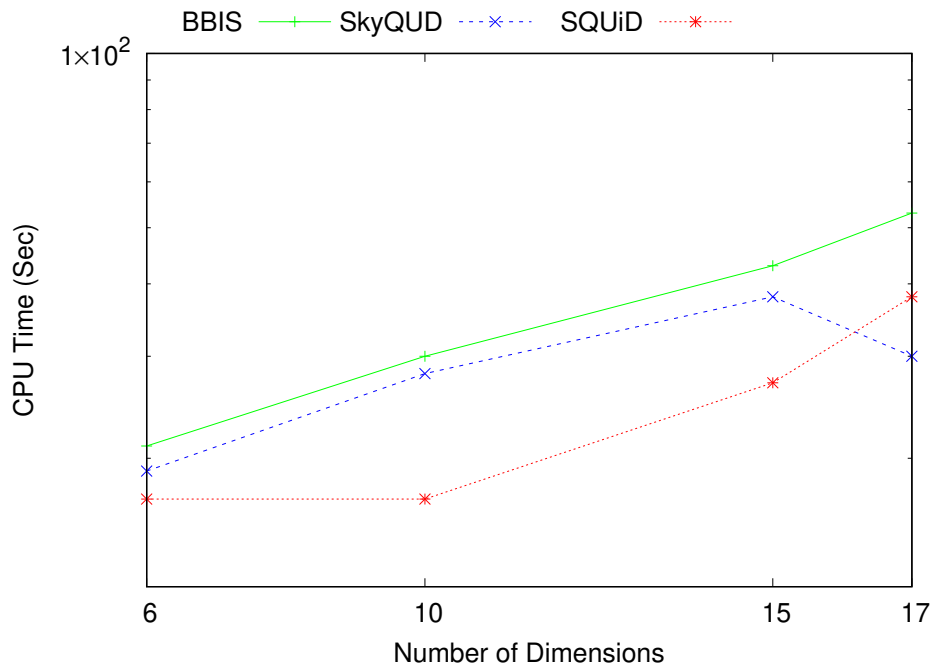

Supplement: Supplemental Information 2 [file peerj-cs-10-2225-s002.zip › Peer-SQUiDExperimentalResults/DM1/DMCPU/outputN-eps-converted-to.pdf]

Number of Pairwise Comparisons

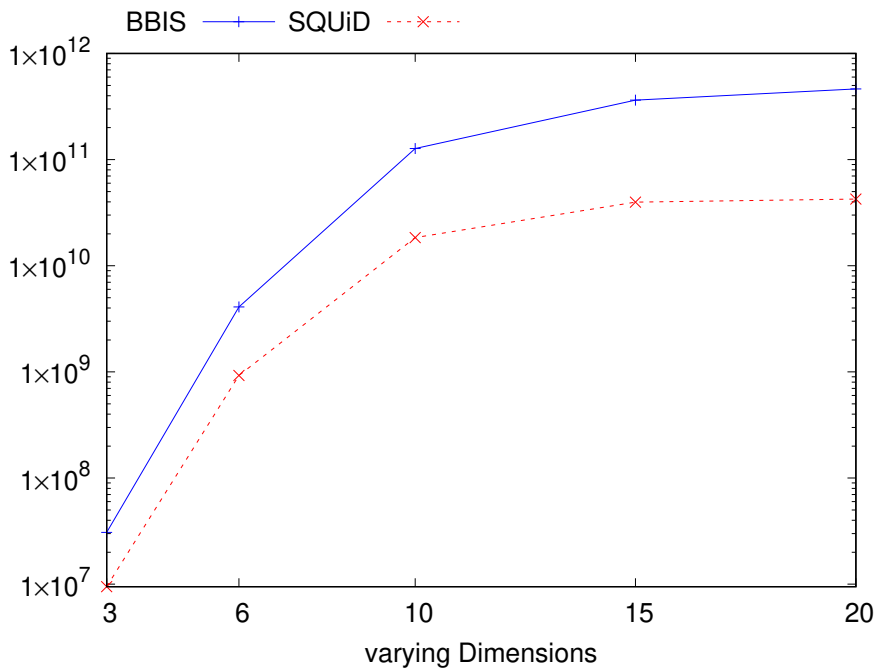

Supplement: Supplemental Information 2 [file peerj-cs-10-2225-s002.zip › Peer-SQUiDExperimentalResults/DM1/DMNNV/outputAC-eps-converted-to.pdf]

Number of Pairwise Comparisons

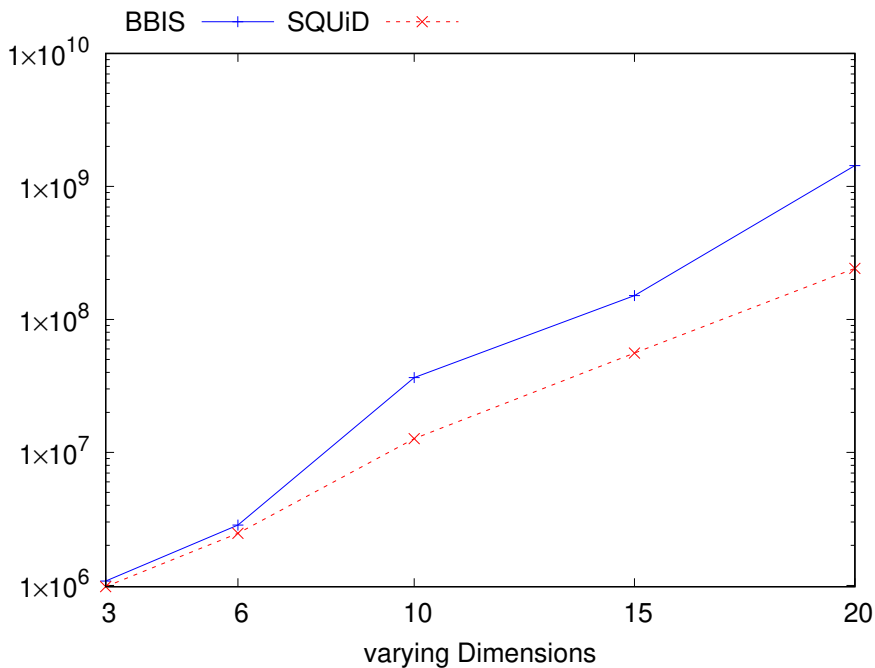

Supplement: Supplemental Information 2 [file peerj-cs-10-2225-s002.zip › Peer-SQUiDExperimentalResults/DM1/DMNNV/outputC-eps-converted-to.pdf]

Number of Pairwise Comparisons

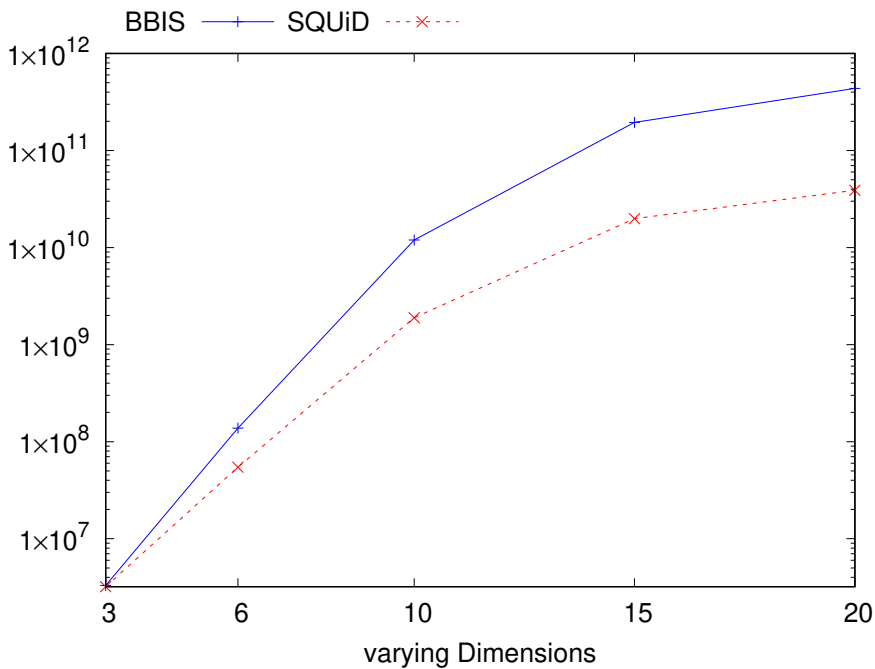

Supplement: Supplemental Information 2 [file peerj-cs-10-2225-s002.zip › Peer-SQUiDExperimentalResults/DM1/DMNNV/outputI-eps-converted-to.pdf]

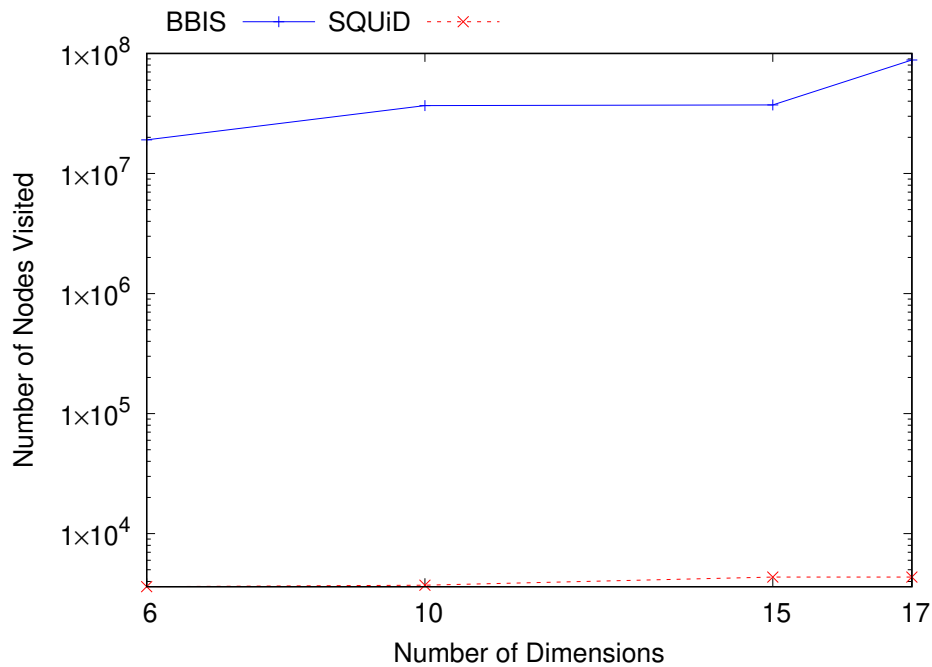

Supplement: Supplemental Information 2 [file peerj-cs-10-2225-s002.zip › Peer-SQUiDExperimentalResults/DM1/DMNNV/outputN-eps-converted-to.pdf]

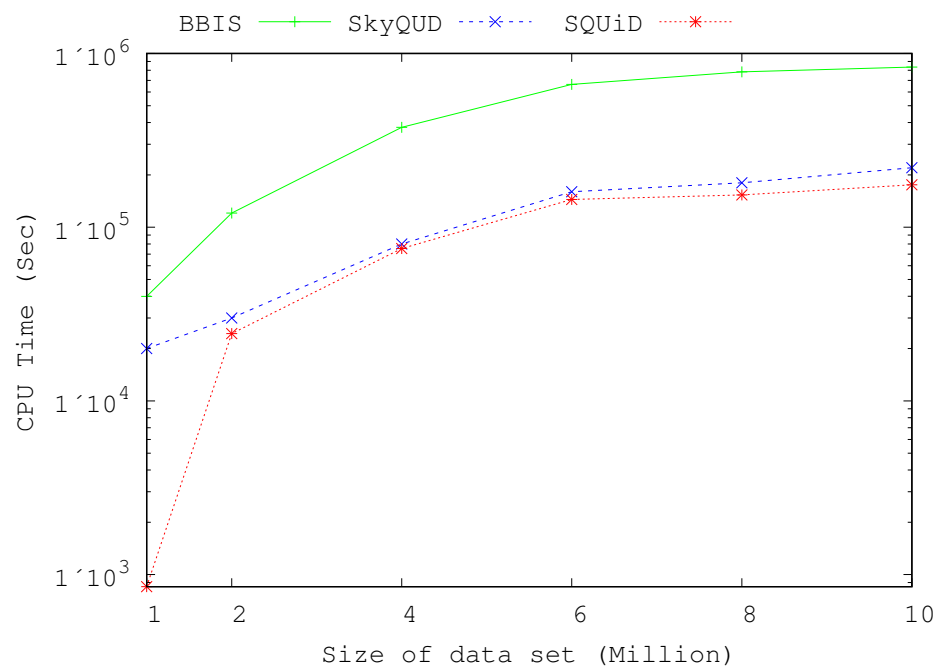

Supplement: Supplemental Information 2 [file peerj-cs-10-2225-s002.zip › Peer-SQUiDExperimentalResults/DS/DSCPU/outputAC-eps-converted-to.pdf]

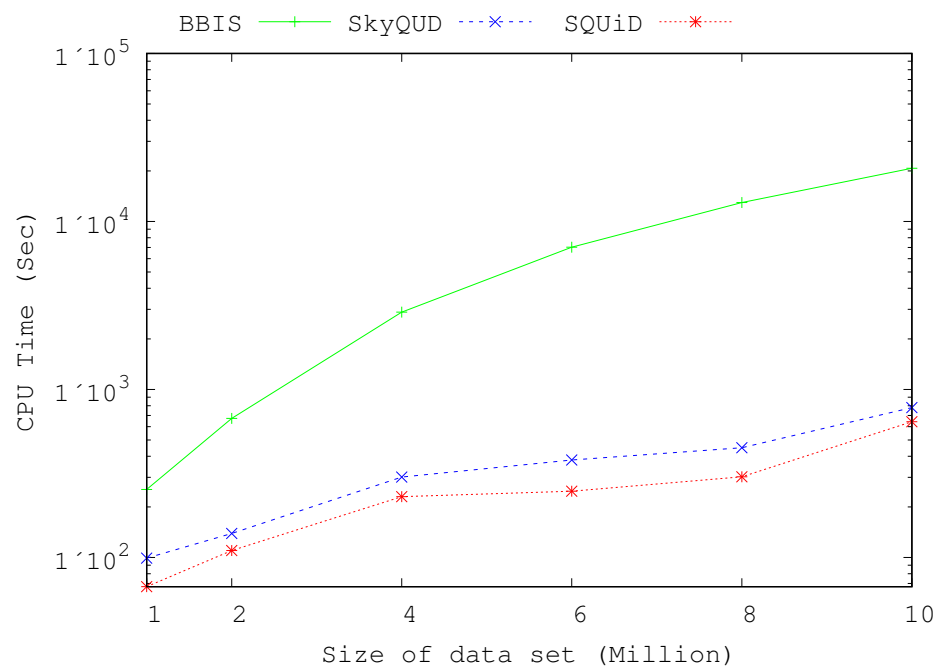

Supplement: Supplemental Information 2 [file peerj-cs-10-2225-s002.zip › Peer-SQUiDExperimentalResults/DS/DSCPU/outputC-eps-converted-to.pdf]

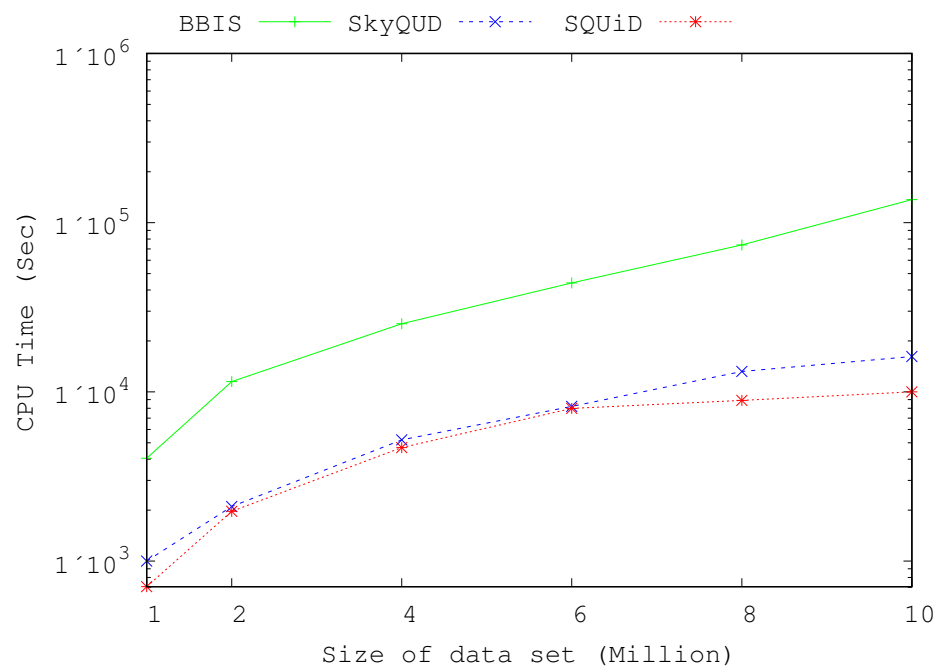

Supplement: Supplemental Information 2 [file peerj-cs-10-2225-s002.zip › Peer-SQUiDExperimentalResults/DS/DSCPU/outputI-eps-converted-to.pdf]

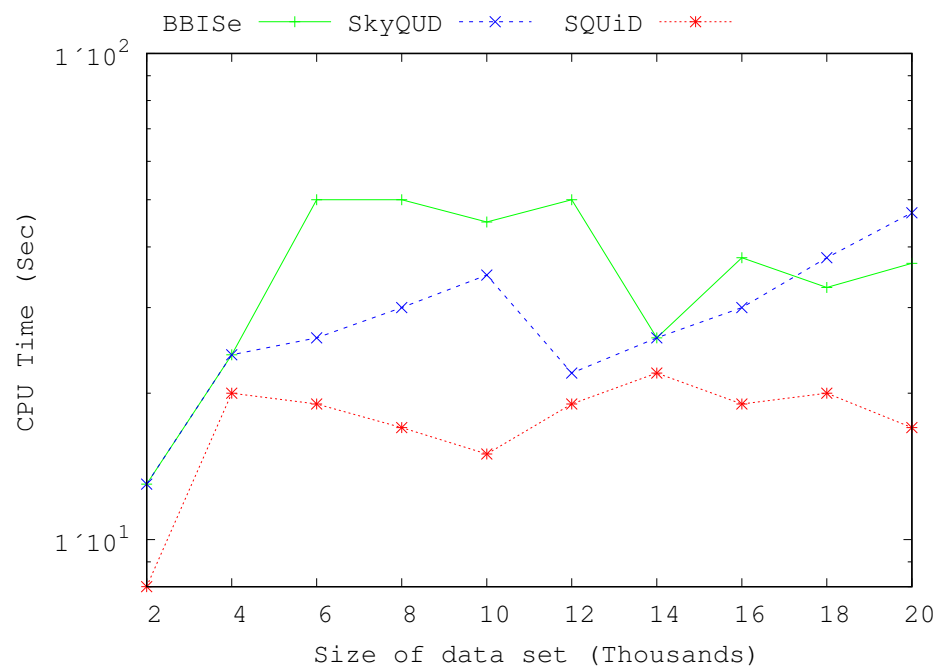

Supplement: Supplemental Information 2 [file peerj-cs-10-2225-s002.zip › Peer-SQUiDExperimentalResults/DS/DSCPU/outputN-eps-converted-to.pdf]

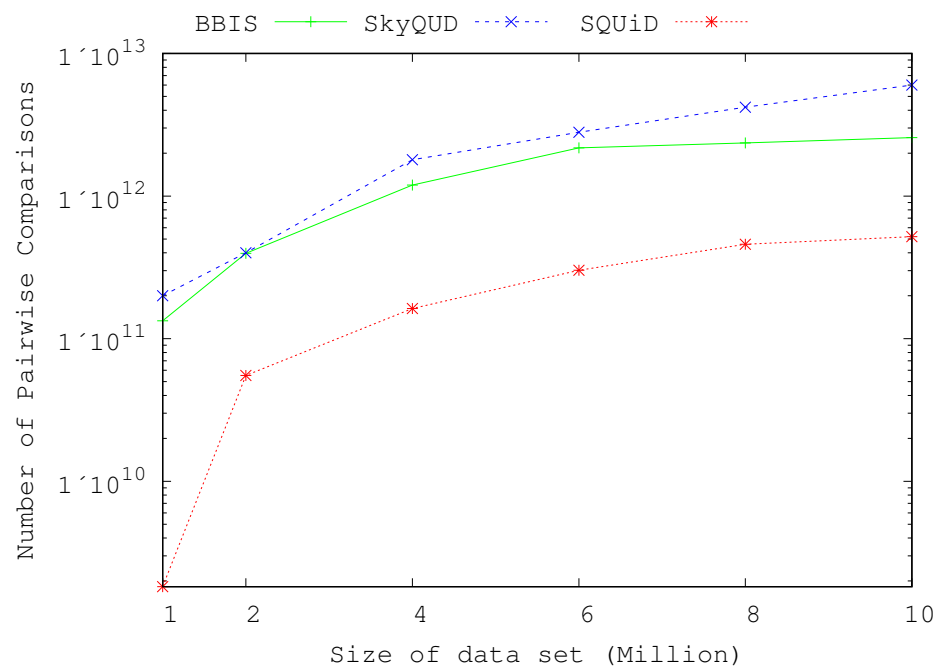

Supplement: Supplemental Information 2 [file peerj-cs-10-2225-s002.zip › Peer-SQUiDExperimentalResults/DS/DSNNV/outputAC-eps-converted-to.pdf]

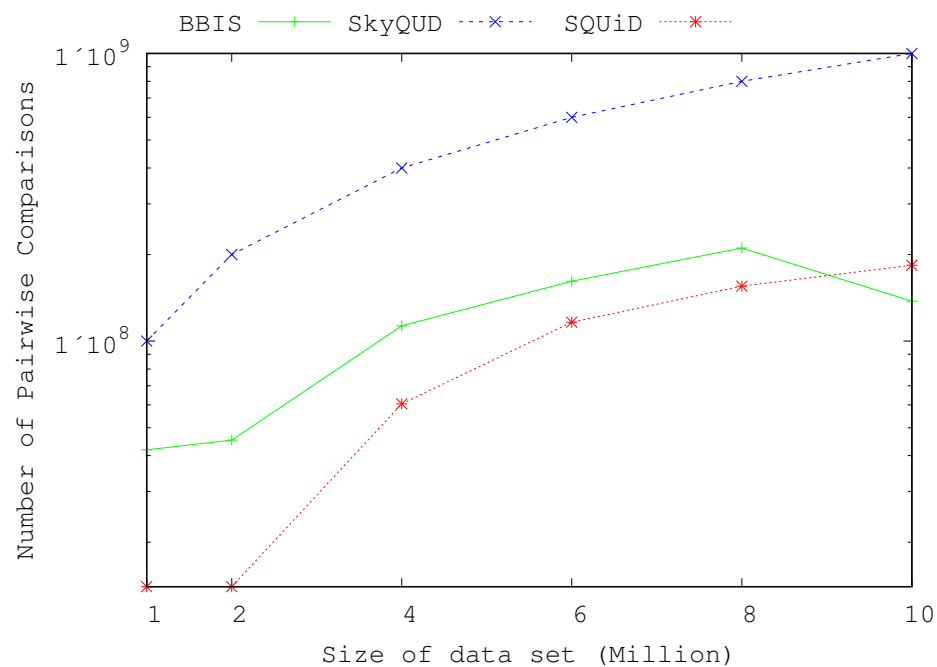

Supplement: Supplemental Information 2 [file peerj-cs-10-2225-s002.zip › Peer-SQUiDExperimentalResults/DS/DSNNV/outputC-eps-converted-to.pdf]

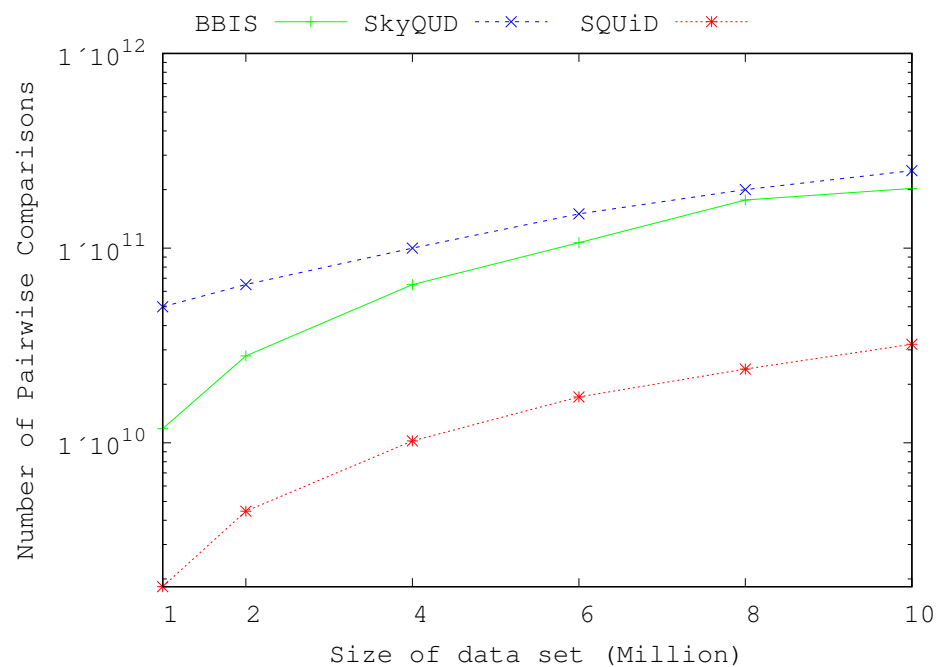

Supplement: Supplemental Information 2 [file peerj-cs-10-2225-s002.zip › Peer-SQUiDExperimentalResults/DS/DSNNV/outputI-eps-converted-to.pdf]

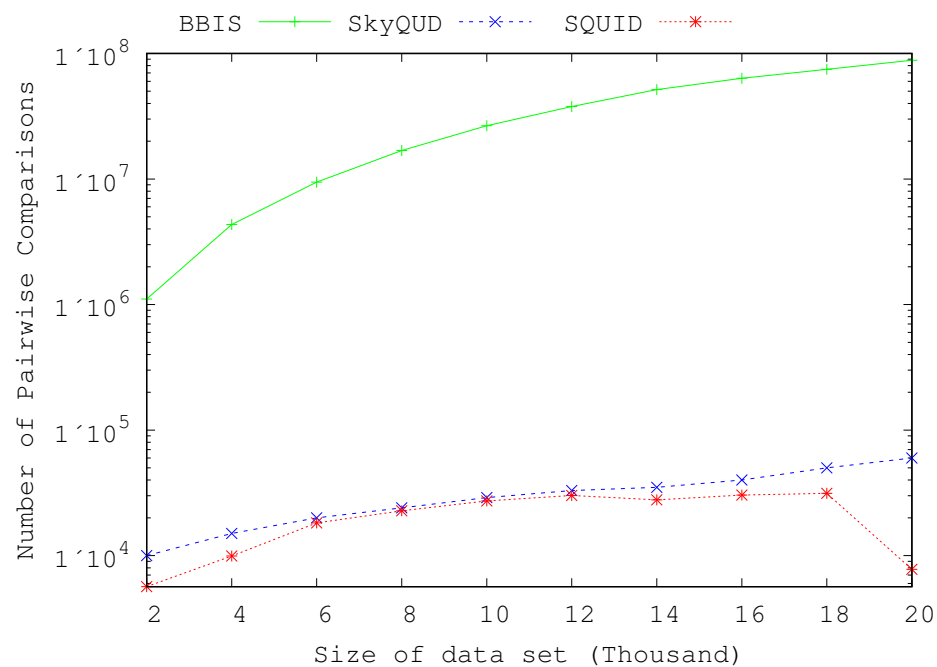

Supplement: Supplemental Information 2 [file peerj-cs-10-2225-s002.zip › Peer-SQUiDExperimentalResults/DS/DSNNV/outputN-eps-converted-to.pdf]

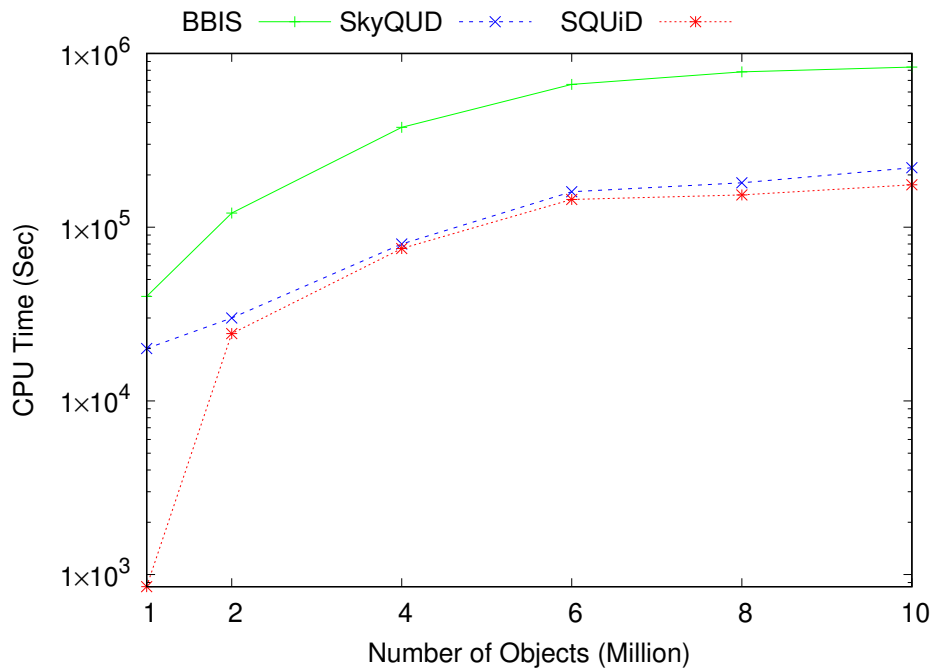

Supplement: Supplemental Information 2 [file peerj-cs-10-2225-s002.zip › Peer-SQUiDExperimentalResults/DS1/DSCPU/outputAC-eps-converted-to.pdf]

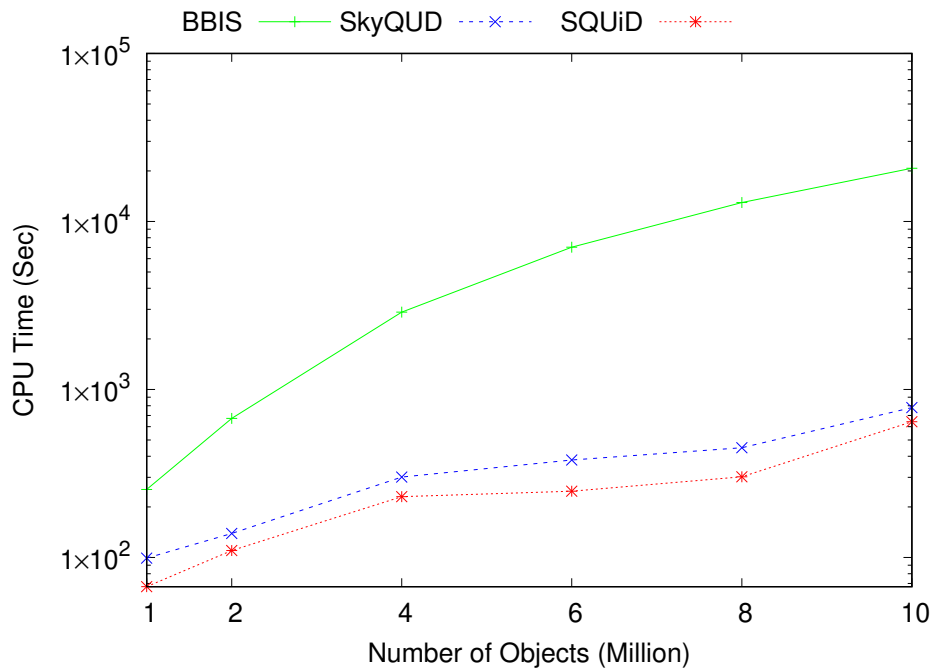

Supplement: Supplemental Information 2 [file peerj-cs-10-2225-s002.zip › Peer-SQUiDExperimentalResults/DS1/DSCPU/outputC-eps-converted-to.pdf]

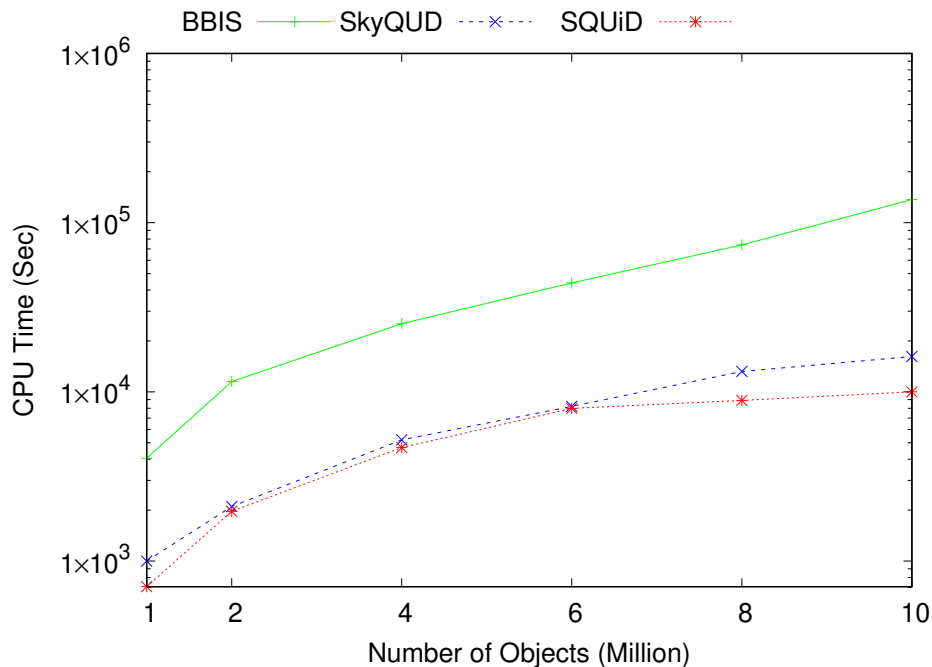

Supplement: Supplemental Information 2 [file peerj-cs-10-2225-s002.zip › Peer-SQUiDExperimentalResults/DS1/DSCPU/outputI-eps-converted-to.pdf]

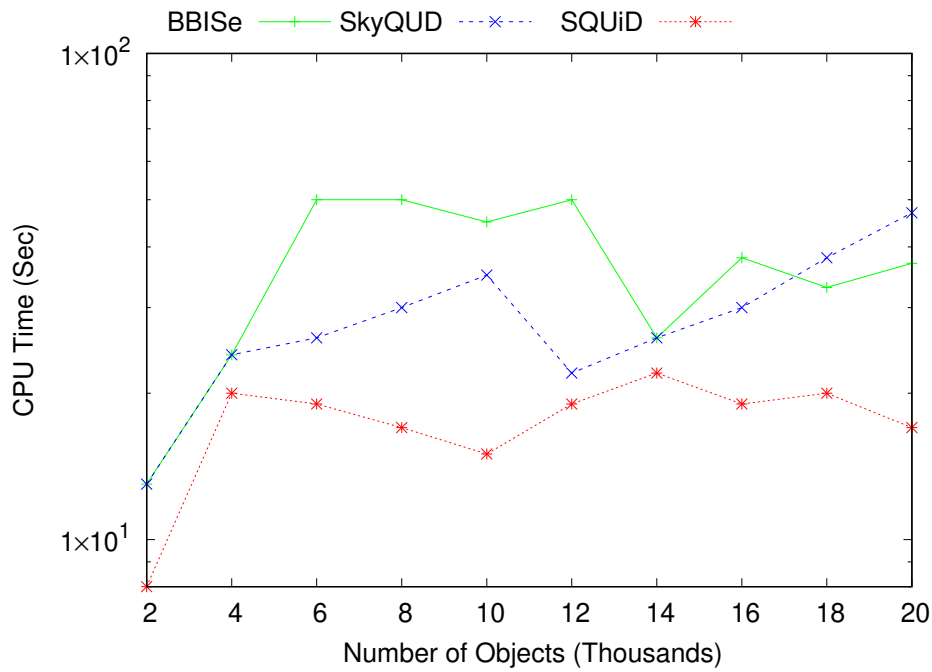

Supplement: Supplemental Information 2 [file peerj-cs-10-2225-s002.zip › Peer-SQUiDExperimentalResults/DS1/DSCPU/outputN-eps-converted-to.pdf]

Number of Pairwise Comparisons

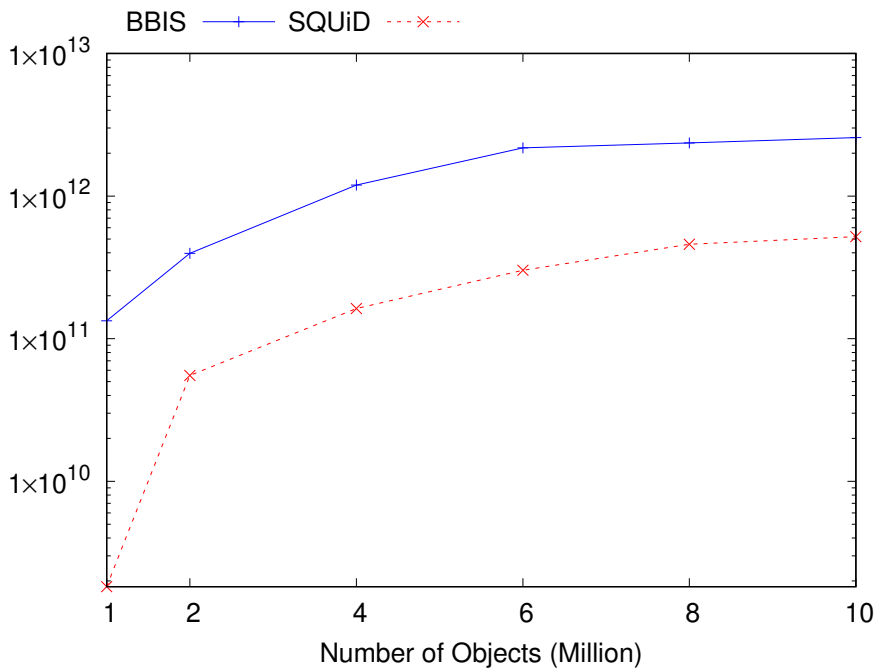

Supplement: Supplemental Information 2 [file peerj-cs-10-2225-s002.zip › Peer-SQUiDExperimentalResults/DS1/DSNNV/outputAC-eps-converted-to.pdf]

Number of Pairwise Comparisons

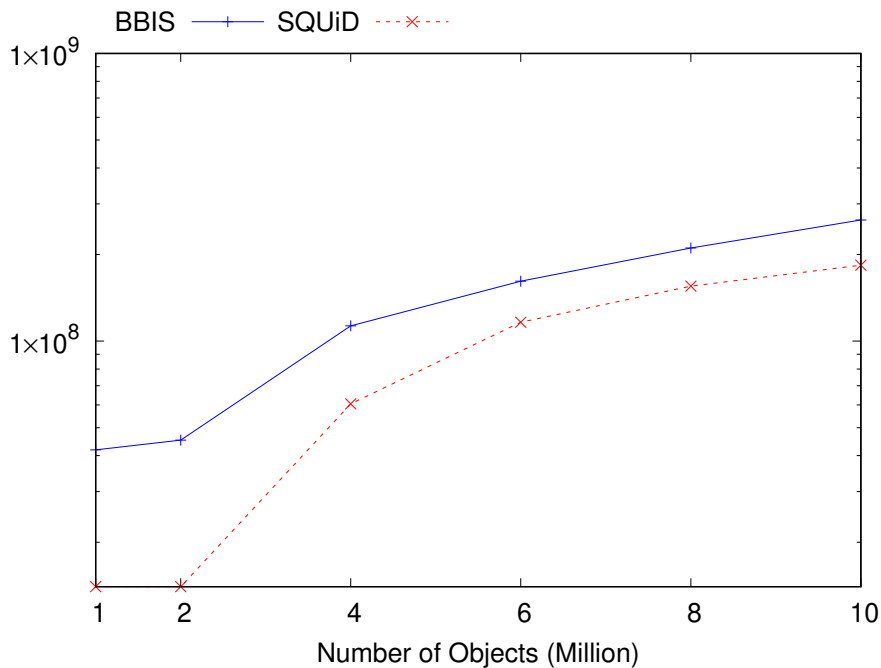

Supplement: Supplemental Information 2 [file peerj-cs-10-2225-s002.zip › Peer-SQUiDExperimentalResults/DS1/DSNNV/outputC-eps-converted-to.pdf]

Number of Pairwise Comparisons

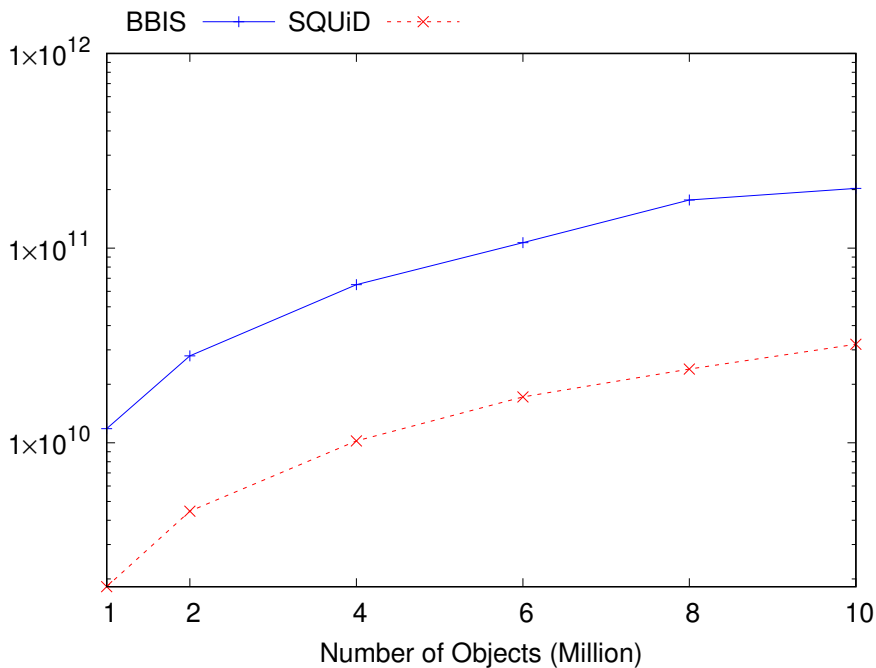

Supplement: Supplemental Information 2 [file peerj-cs-10-2225-s002.zip › Peer-SQUiDExperimentalResults/DS1/DSNNV/outputI-eps-converted-to.pdf]

Number of Pairwise Comparisons

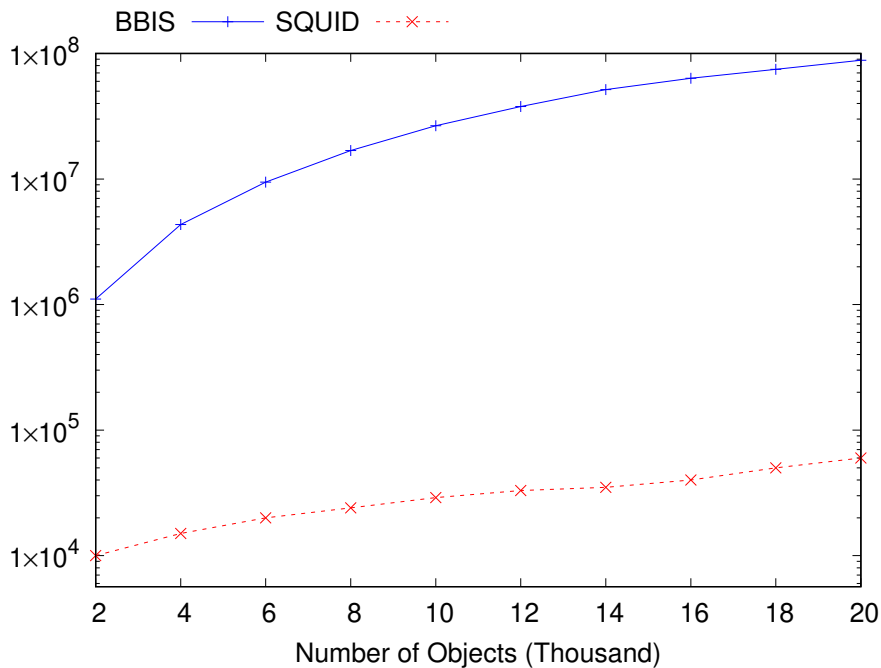

Supplement: Supplemental Information 2 [file peerj-cs-10-2225-s002.zip › Peer-SQUiDExperimentalResults/DS1/DSNNV/outputN-eps-converted-to.pdf]

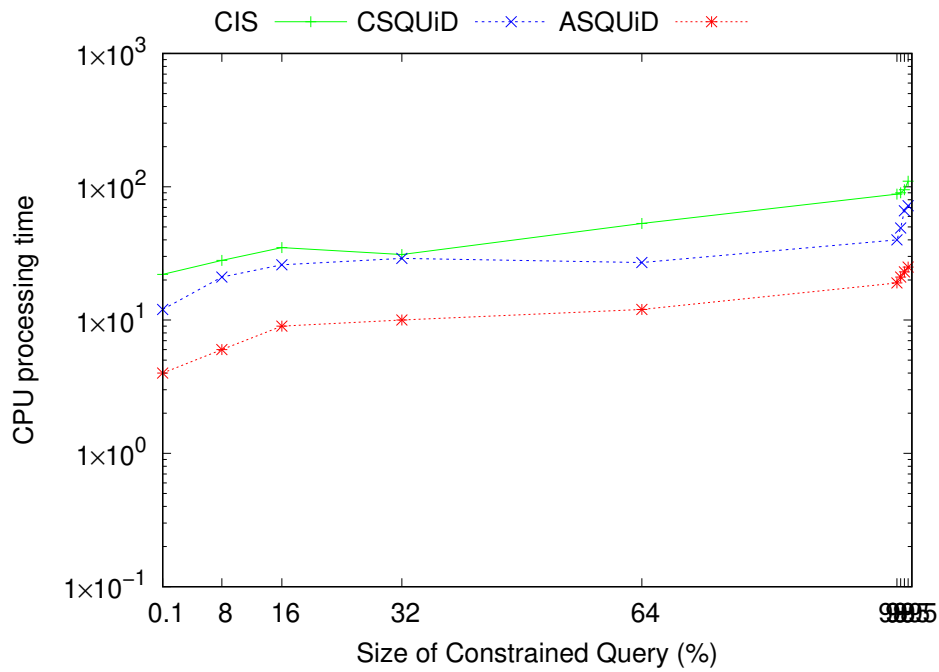

Supplement: Supplemental Information 2 [file peerj-cs-10-2225-s002.zip › Peer-SQUiDExperimentalResults/MV/MV2/outputAC-eps-converted-to.pdf]

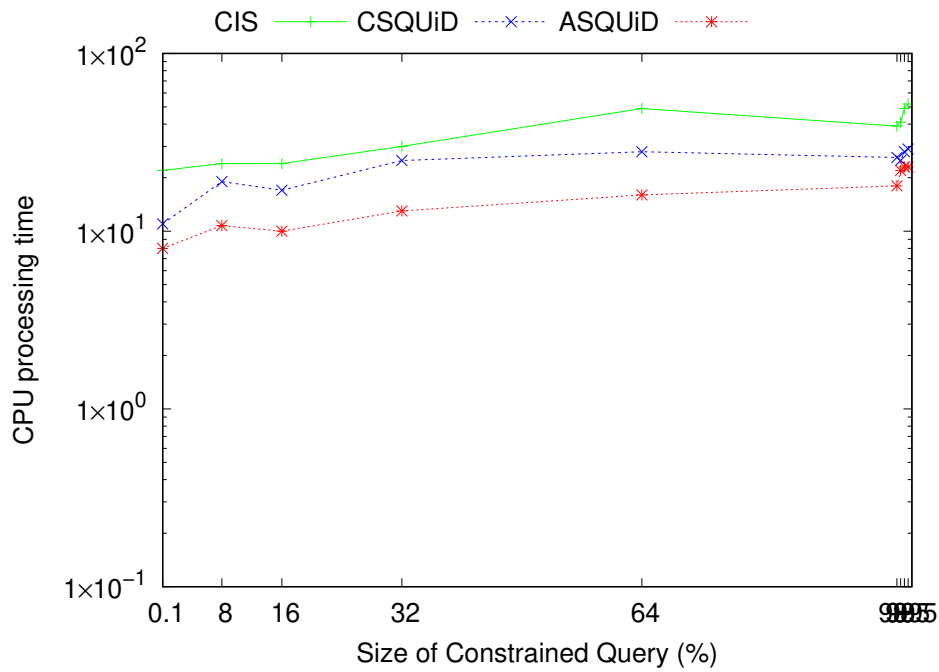

Supplement: Supplemental Information 2 [file peerj-cs-10-2225-s002.zip › Peer-SQUiDExperimentalResults/MV/MV2/outputC-eps-converted-to.pdf]

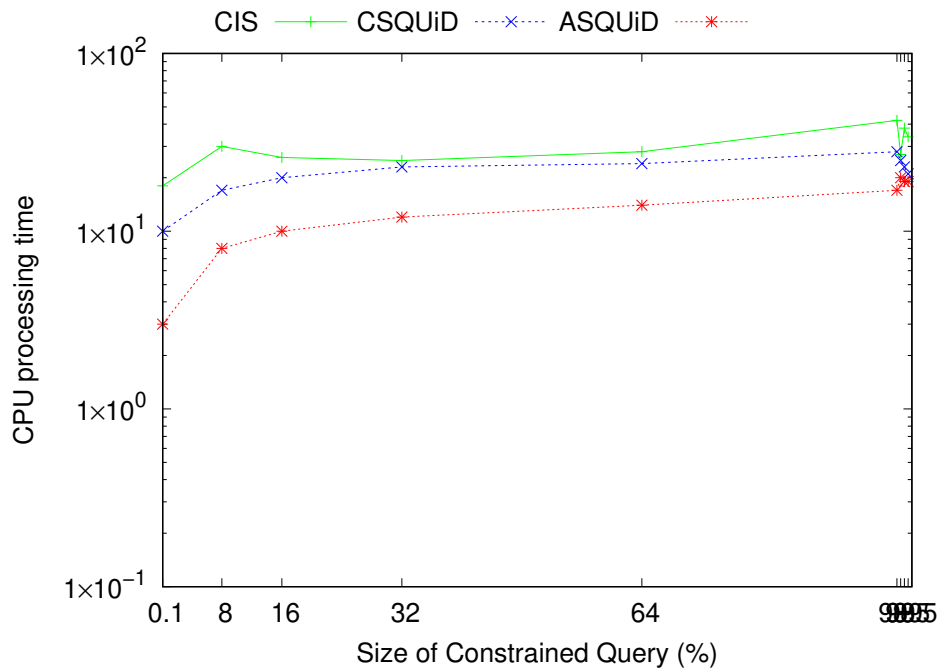

Supplement: Supplemental Information 2 [file peerj-cs-10-2225-s002.zip › Peer-SQUiDExperimentalResults/MV/MV2/outputI-eps-converted-to.pdf]

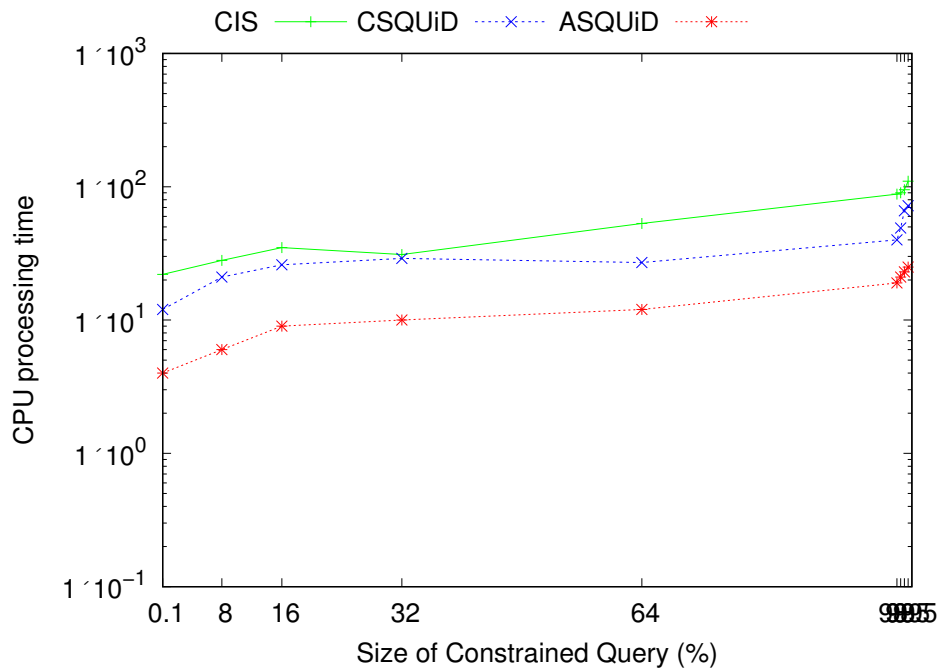

Supplement: Supplemental Information 2 [file peerj-cs-10-2225-s002.zip › Peer-SQUiDExperimentalResults/MV/MVCPU/outputAC-eps-converted-to.pdf]

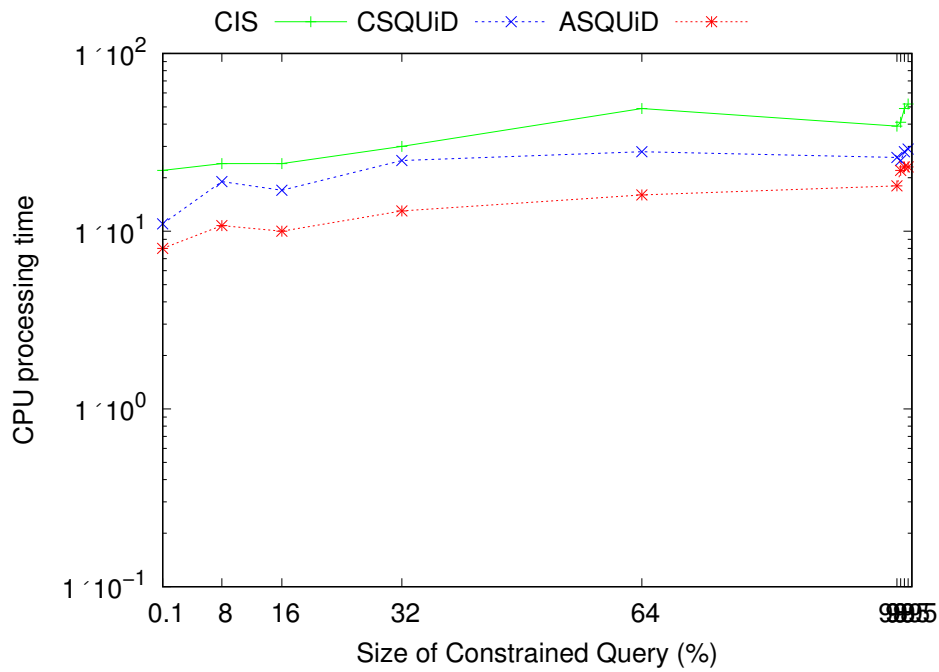

Supplement: Supplemental Information 2 [file peerj-cs-10-2225-s002.zip › Peer-SQUiDExperimentalResults/MV/MVCPU/outputC-eps-converted-to.pdf]

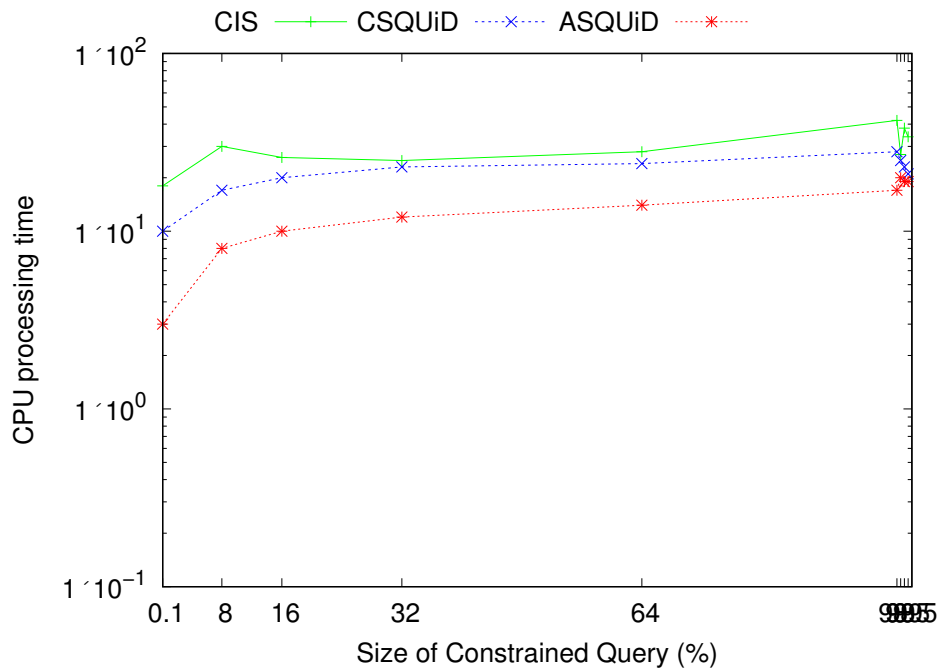

Supplement: Supplemental Information 2 [file peerj-cs-10-2225-s002.zip › Peer-SQUiDExperimentalResults/MV/MVCPU/outputI-eps-converted-to.pdf]

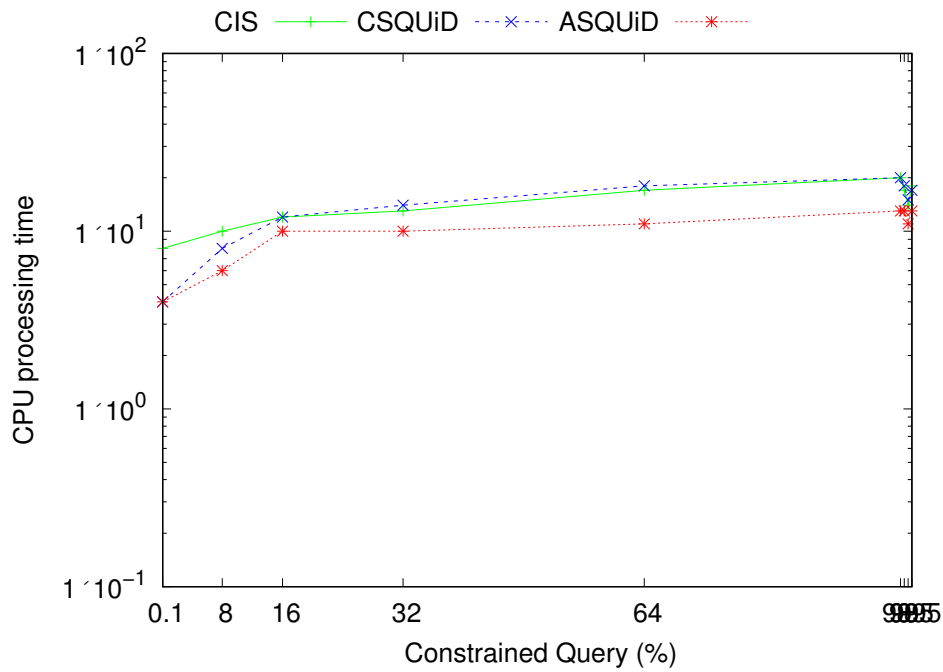

Supplement: Supplemental Information 2 [file peerj-cs-10-2225-s002.zip › Peer-SQUiDExperimentalResults/MV/MVCPU/outputN-eps-converted-to.pdf]

Number of Pairwise Comparisons

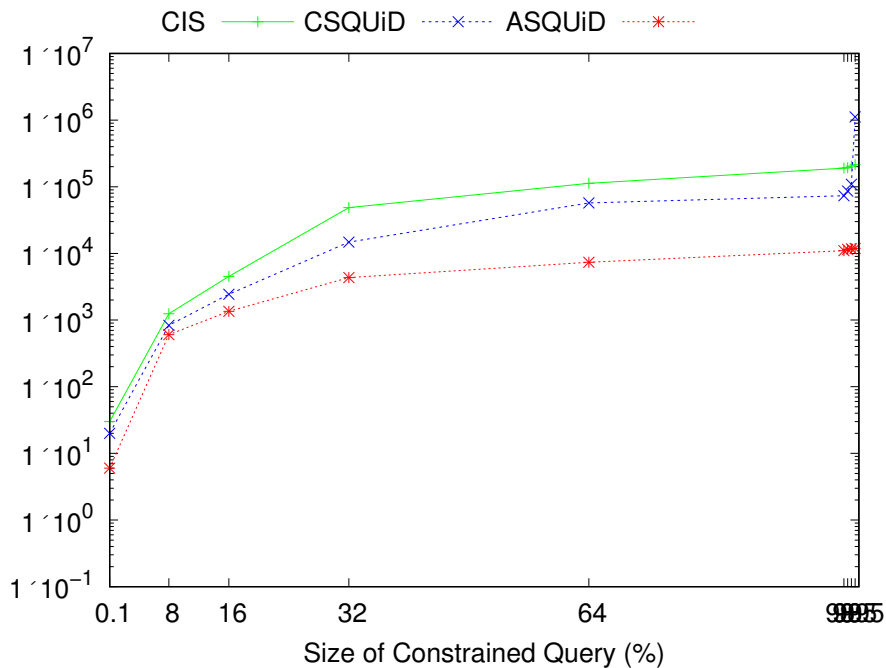

Supplement: Supplemental Information 2 [file peerj-cs-10-2225-s002.zip › Peer-SQUiDExperimentalResults/MV/MVNNV/outputAC-eps-converted-to.pdf]

Number of Pairwise Comparisons

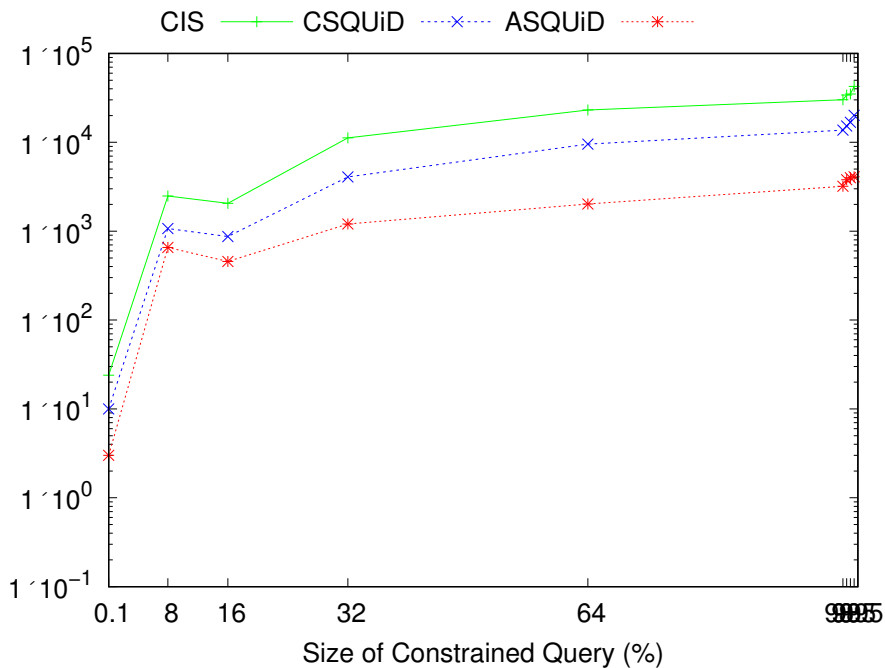

Supplement: Supplemental Information 2 [file peerj-cs-10-2225-s002.zip › Peer-SQUiDExperimentalResults/MV/MVNNV/outputC-eps-converted-to.pdf]

Number of Pairwise Comparisons

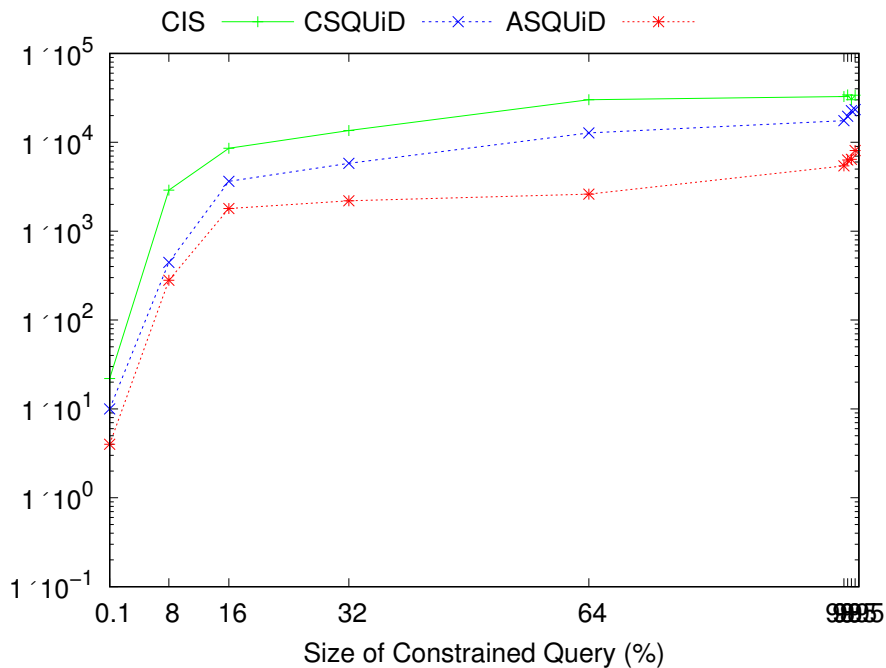

Supplement: Supplemental Information 2 [file peerj-cs-10-2225-s002.zip › Peer-SQUiDExperimentalResults/MV/MVNNV/outputI-eps-converted-to.pdf]

Number of Pairwise Comparisons

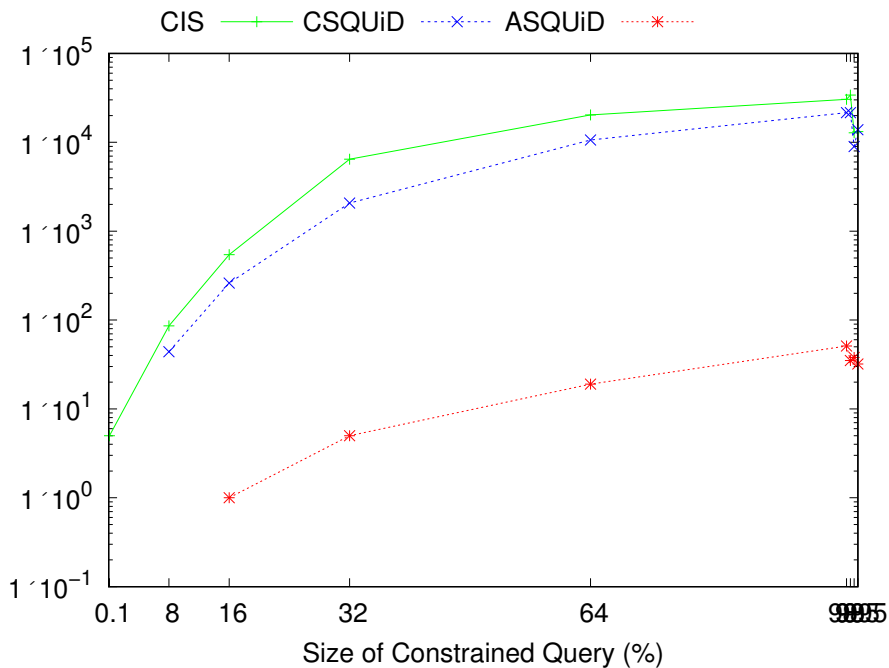

Supplement: Supplemental Information 2 [file peerj-cs-10-2225-s002.zip › Peer-SQUiDExperimentalResults/MV/MVNNV/outputN-eps-converted-to.pdf]
